# Reduced insulin signaling maintains electrical transmission in a neural circuit in aging flies

**DOI:** 10.1371/journal.pbio.2001655

**Published:** 2017-09-13

**Authors:** Hrvoje Augustin, Kieran McGourty, Marcus J. Allen, Sirisha Kudumala Madem, Jennifer Adcott, Fiona Kerr, Chi Tung Wong, Alec Vincent, Tanja Godenschwege, Emmanuel Boucrot, Linda Partridge

**Affiliations:** 1 Max Planck Institute for Biology of Aging, Köln, Germany; 2 Institute of Healthy Aging, and Genetics, Evolution, and Environment, University College London, London, United Kingdom; 3 Department of Structural and Molecular Biology, London, United Kingdom; 4 School of Biosciences, University of Kent, Canterbury, Kent, United Kingdom; 5 Department of Biological Sciences, Florida Atlantic University, Jupiter, Florida, United States of America; Princeton University, United States of America

## Abstract

Lowered insulin/insulin-like growth factor (IGF) signaling (IIS) can extend healthy lifespan in worms, flies, and mice, but it can also have adverse effects (the “insulin paradox”). Chronic, moderately lowered IIS rescues age-related decline in neurotransmission through the *Drosophila* giant fiber system (GFS), a simple escape response neuronal circuit, by increasing targeting of the gap junctional protein innexin shaking-B to gap junctions (GJs). Endosomal recycling of GJs was also stimulated in cultured human cells when IIS was reduced. Furthermore, increasing the activity of the recycling small guanosine triphosphatases (GTPases) Rab4 or Rab11 was sufficient to maintain GJs upon elevated IIS in cultured human cells and in flies, and to rescue age-related loss of GJs and of GFS function. Lowered IIS thus elevates endosomal recycling of GJs in neurons and other cell types, pointing to a cellular mechanism for therapeutic intervention into aging-related neuronal disorders.

## Author summary

Insulin and insulin-like growth factors play an important role in the nervous system development and function. Reduced insulin signaling, however, can improve symptoms of neurodegenerative diseases in different model organisms and protect against age-associated decline in neuronal function extending lifespan. Here, we analyze the effects of genetically attenuated insulin signaling on the escape response pathway in the fruit fly *Drosophila melanogaster*. This simple neuronal circuit is dominated by electrical synapses composed of the gap junctional shaking-B protein, which allows for the transfer of electrical impulses between cells. Transmission through the circuit is known to slow down with age. We show that this functional decline is prevented by systemic or circuit-specific suppression of insulin signaling due to the preservation of the number of gap junctional proteins in aging animals. Our experiments in a human cell culture system reveal increased membrane targeting of gap junctional proteins via small proteins Rab4 and Rab11 under reduced insulin conditions. We also find that increasing the level of these recycling-mediating proteins in flies preserves the escape response circuit output in old flies and suggests ways of improving the function of neuronal circuits dominated by electrical synapses during aging.

## Introduction

Synapses undergo age-associated morphological and functional changes in a number of model organisms [1–3] and in humans [4–6]. In the fruit fly *Drosophila melanogaster* and the worm *Caenorhabditis elegans*, synaptic changes were seen during normal aging in both central and peripheral parts of the nervous system and linked to cognition, memory, learning, locomotor, and homeostatic deficits [7–14].

In neurons, gap junctions (GJs) constitute the morphological substrate of electric (i.e., electrotonic) synapses characterized by electrical coupling, and permeability for small molecules generally up to approximately 1 kDa (i.e., metabolic or biochemical coupling). GJs play vital roles in the distribution of metabolic substrates [15], tissue development, homeostasis [16], and cell-to-cell communication via calcium waves [17]. Electrical synapses serve important functions in the sensory and motor neurons [18], and in learning and memory [19]. Gap junctional communication is also critical for (nonexcitable) glial cells, providing a pathway that contributes to the uptake of ions and the release of neuroactive substances, so called “gliotransmitters” [20]. A previous work demonstrated a significant decline with age in the density of connexins Cx43 and Cx30, the most abundant astrocytic gap junctional proteins, in mouse brains [21]. The decline in function of the GJs with age is likely to have a pervasive role in both invertebrate and vertebrate nervous systems because electrical synapses play a major role in both [18]. Considering that 25% of the fruit fly brain is composed of glial cells, while in the human brain this percentage reaches about 90% [22], the importance of GJs for nervous system function is profound.

Reduced insulin/insulin-like growth factor (IGF) signaling (IIS) can ameliorate the effects of aging in multiple model organisms and, probably, humans [23,24], pointing the way to a broad-spectrum, preventative medicine for the diseases of human aging. The nervous system is an important case in point, as both insulin and IGF are expressed across physiologically distinct brain regions [25,26] and have well known roles in the development, growth, and survival of the central nervous system [27]. In addition, lowered IIS can lead to insulin resistance and diabetes, neuronal injury [28], and compromised neural control of metabolism [29]. Despite this, chronically lowered insulin/IGF signaling can improve metabolic, synaptic, and cognitive defects in rodent and *Drosophila* models of several neurodegenerative diseases [30–36], leading to what has become known as the “insulin paradox” [37]. The molecular mechanisms through which lowered IIS mediates improved health, particularly in the nervous system [37,38] are poorly understood.

Here, we chose to study electrical transmission in the giant fiber system (GFS), the fly’s escape response neuronal circuit. Electrical synapses in the GFS are formed by the products of the shaking-B gene, *SHAK-B* [39] and represent the dominant synapse type in the circuit. Conduction through the GFS in *SHAK-B* mutants is very weak [40,41], with individual animals producing either no response or a significantly delayed response to a stimulus. In addition, when the chemical (cholinergic) synapses are disabled with tetanus toxin, the circuit function is largely unaffected [42], further demonstrating the predominantly “electrical” nature of this neuronal system. We showed that IIS silencing improves function of the electrical component of the GFS in aging flies by preventing the loss of the principal gap junctional shaking-B protein (SHAK-B). This preservation of the GJ density and circuit function is likely mediated by the recycling-promoting Rab4 and Rab11 proteins. Our experiments in human cells showed increased lysosomal targeting with elevated IIS, with IIS reduction and Rab4/11 over-expression resulting in increased density of GJs.

## Results

### Ubiquitously reduced IIS prevents age-related functional decline of the *Drosophila* escape response circuit

**Fig 1:**
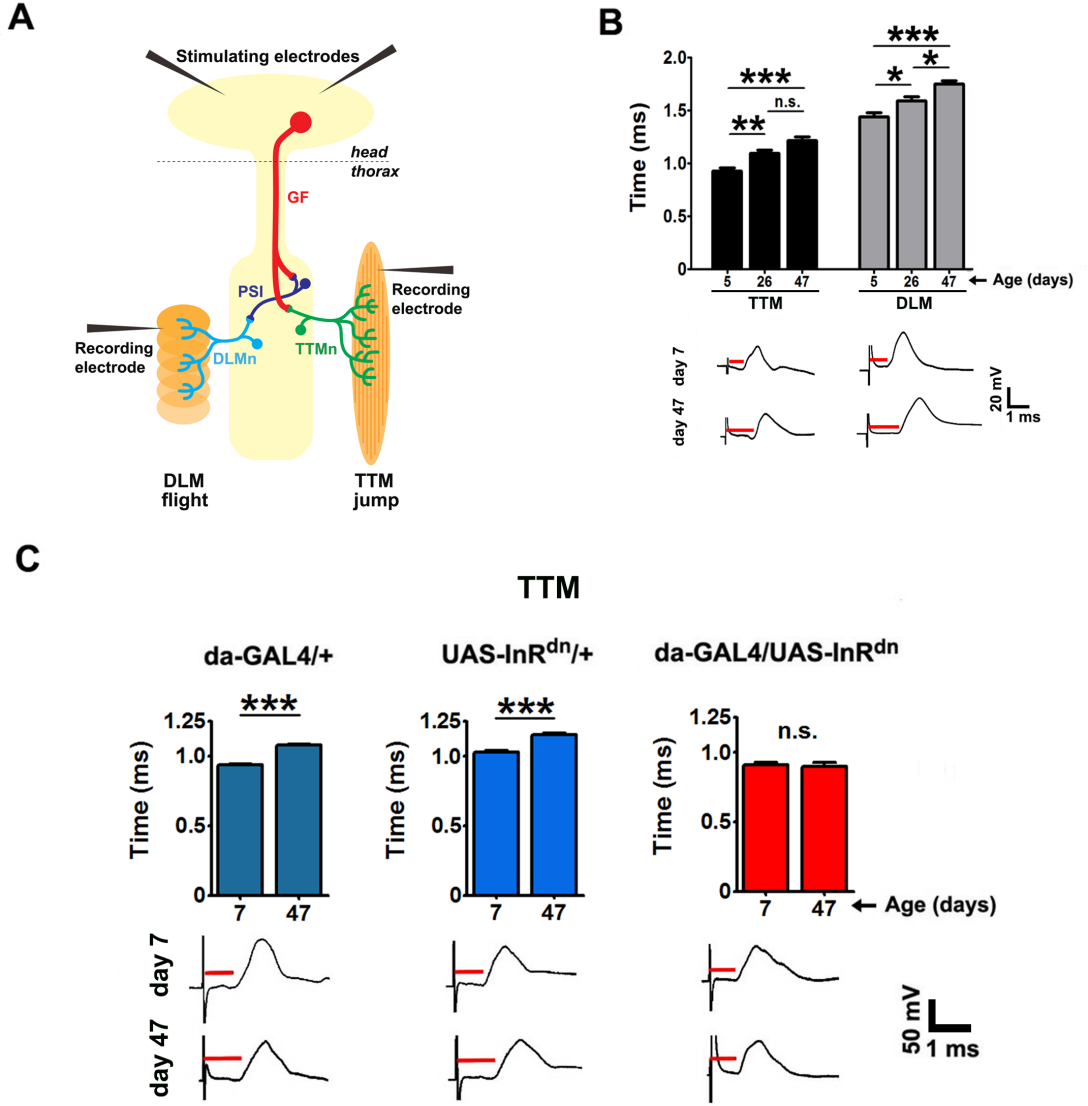
Ubiquitously reduced insulin/insulin-like growth factor signaling (IIS) prevents age-associated decline in transmission through the giant fiber system (GFS). (A) The GFS, showing insertion sites for recording and stimulating electrodes. The monosynaptic tergotrochanteral muscle (TTM) pathway involves the large electrochemical giant fiber (GF)- tergotrochanteral muscle motor neuron (TTMn) synapse. The electrochemical GF-peripherally synapsing interneurons (PSI), PSI, and chemical (cholinergic) PSI-dorsal longitudinal muscle motor neuron (DLMn) synapses comprise the bisynaptic dorsal longitudinal muscle (DLM) pathway. All neuromuscular synapses are chemical (glutamatergic). (B) Response latencies significantly increased with age when recorded from the TTMs (left) and DLMs (right) (*n* = 8–9). Representative TTM and DLM traces are shown below. Red bars indicate the time between brain stimulus and muscle response. (C) Reduced IIS (*da-GAL4/UAS-InR*^*dn*^) prevented age-associated decline in GFS transmission (age x genotype interaction between the control genotypes and *da-GAL4/UAS-InR*^*dn*^ is significant, *P* < 0.03; *n* = 6–16; S1 Table). Error bars denote SEM.

The bilaterally symmetrical GFS (Fig 1A), a well-characterized, multicomponent neuronal circuit [43,44], mediates fast escape behavior by extension of the legs [45] followed by flight. It consists of 2 giant fiber (GF) interneurons that descend from the brain and synapse in the thoracic neuromere both with peripherally synapsing interneurons (PSI), which in turn synapse with dorsal longitudinal flight muscle motor neurons (DLMns) innervating the dorsal longitudinal flight muscles (DLMs), and with motor neurons (tergotrochanteral muscle motor neuron [TTMn]) innervating the tergotrochanteral (jump) muscles (TTMs). Electrical brain stimulation activates the GF interneurons, and the 2 output pathways can be monitored by recording from the 2 muscles (Fig 1A). Rapid conduction of nerve impulses through the escape response pathway has survival value [46]. Response latency, the time between the brain stimulus and muscle response, is a reliable measure of GF conduction velocity and circuit functionality [39,42,47].

To assess aging of the GFS, we measured response latencies in wild-type (WT; *w*^*Dah*^) flies at different adult ages (old flies are defined as ≥45 days old). At day 5, the latencies were similar to those previously reported for young WT flies [39,48], and then increased, in both TTMs and DLMs (Fig 1B), indicating that aging slowed transmission in the circuit.

We then assessed whether reduced IIS affected transmission, by using *da-GAL4* driver [49] to ubiquitously and constitutively express a dominant-negative (DN) form of insulin receptor (*InR*^*dn*^) [50], previously shown to extend fly lifespan [51]. Reduced IIS has also been shown to have a positive effect on an olfactory neuronal circuit and odor-related behavior in young flies [52]. The extension of response latency with age, seen in both control groups, was abolished by lowered IIS both in the TTM (Fig 1C) and DLM branch of the pathway (S1A Fig). We also assessed transmission through the TTMn-TTM and DLMn-DLM neuromuscular junctions by directly stimulating the GFS motoneurons of young and old flies. Response latencies of all genotypes were similar to each other and to those previously measured in WT flies [42,44] and were unaffected by fly age (S1B Fig); lowered IIS thus probably acted upstream of the motoneurons and neuromuscular junctions (NMJs) to improve electrical transmission in the aging GFS.

**Fig 2:**
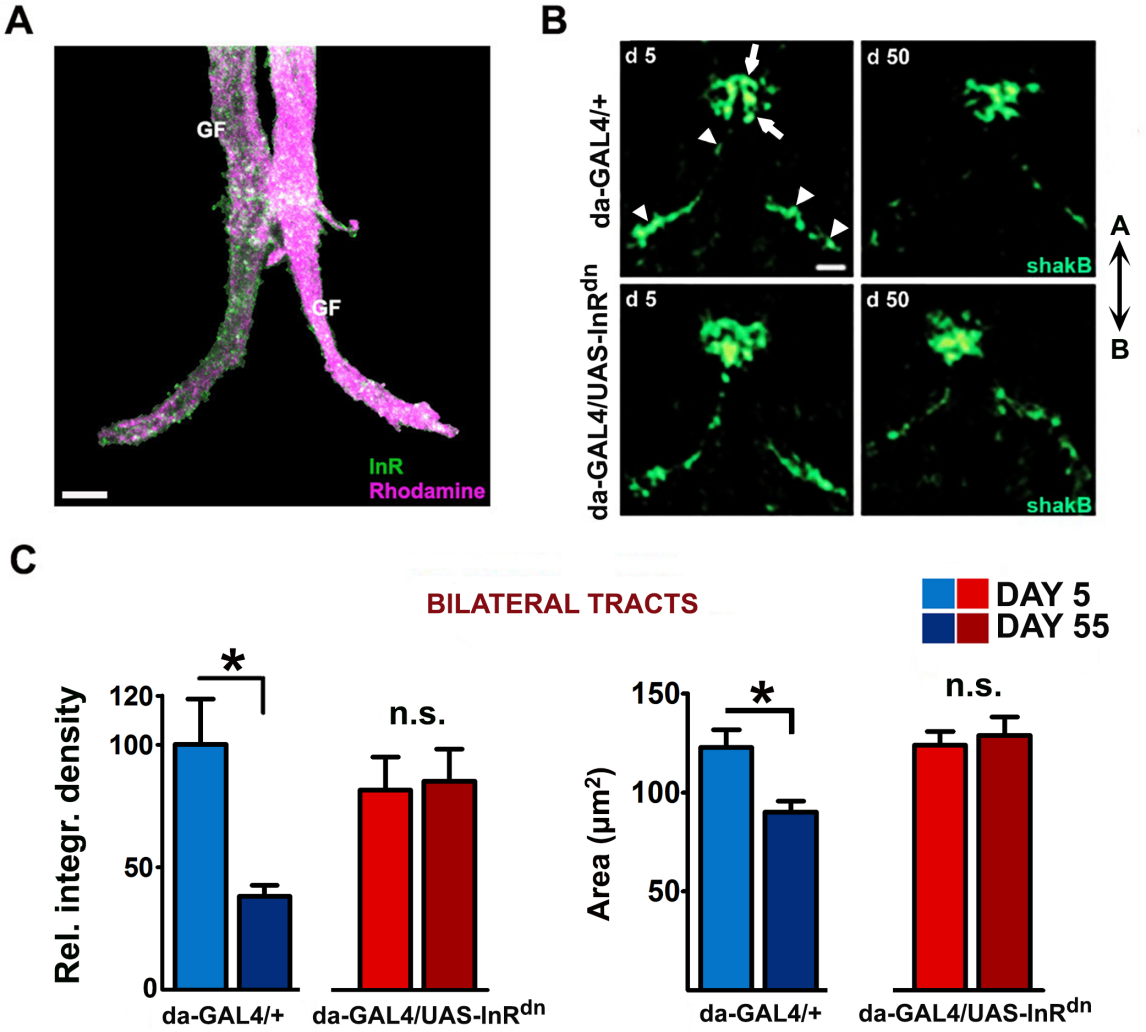
Reduced insulin/insulin-like growth factor signaling (IIS) prevents age-associated loss of gap junctions (GJs) in the giant fiber system (GFS). (A) Insulin receptor (IR) immunolabeling in the giant fiber (GF) interneurons. The 5-day-old GFs were injected with rhodamine-dextran (magenta). Scale bar: 8 μm. (B) Representative confocal images (z-series projections) of thoracic regions enriched in GJs. (Shaking-B protein [SHAK-B] staining): anterior midline area (arrow) and bilateral tracts (arrowheads). The scale bar represents 10 μm for all panels. A and B denote anterior and posterior directions. (C) Quantification of SHAK-B signal intensities (left) and area (right) in the thoracic bilateral tracts (*n* = 5–6 per genotype per age; age x genotype interaction for integrated density: *P* = 0.0328). Error bars denote SEM.

Prompted by these findings, we next asked whether the *Drosophila* insulin receptor (IR) is expressed in the GFS. Indeed, the antiserum previously shown to immunolabel IRs in abdominal neurons [53] detected strong IR presence in the GF interneurons (Fig 2A), allowing for the possibility that GFS is modulated by *Drosophila* insulin-like proteins. To identify mechanisms by which constitutively and ubiquitously reduced IIS maintained GFS function during aging, we assessed the density of electrical synapses, which are assembled from the GJ proteins encoded by *SHAK-B*, and which belong to the innexin family of transmembrane proteins [40,54,55]. In *SHAK-B* mutants, transmission in both the GF-TTM and GF-PSI-DLM pathways is disrupted [39,42]. SHAK-B proteins concentrate in 2 regions of the mesothoracic neuromere: the midline and 2 more posterior bilateral tracts [54] (Fig 2B). We quantified anti-SHAK-B staining intensity and area in isolated central nervous system preparations (that comprise the brain and ventral nerve cord), and found marked reductions in the bilateral tracts (Fig 2C) that label GJs at the GF-TTMn synapse (S2A Fig), potentially explaining the preservation of GFS functionality in *da-GAL4/UAS-InR*^*dn*^ flies.

We also quantified choline acetyltransferase (a marker for cholinergic neurons) and a subunit (α7) of *Drosophila* nicotinic acetylcholine receptors (nAChRs) [56] in immunoblots from isolated central nervous system preparations and from heads but found no difference in immunoreactivity between the young and old IIS mutants or controls (S2B Fig), consistent with absence of structural changes at the level of cholinergic synapses with age. Together with previously mentioned physiological experiments [40–42], these results indicate that longer latencies in aging flies are a consequence of SHAK-B deficiency.

### Neuronal IIS silencing preserves GFS function and gap junctional density

**Fig 3:**
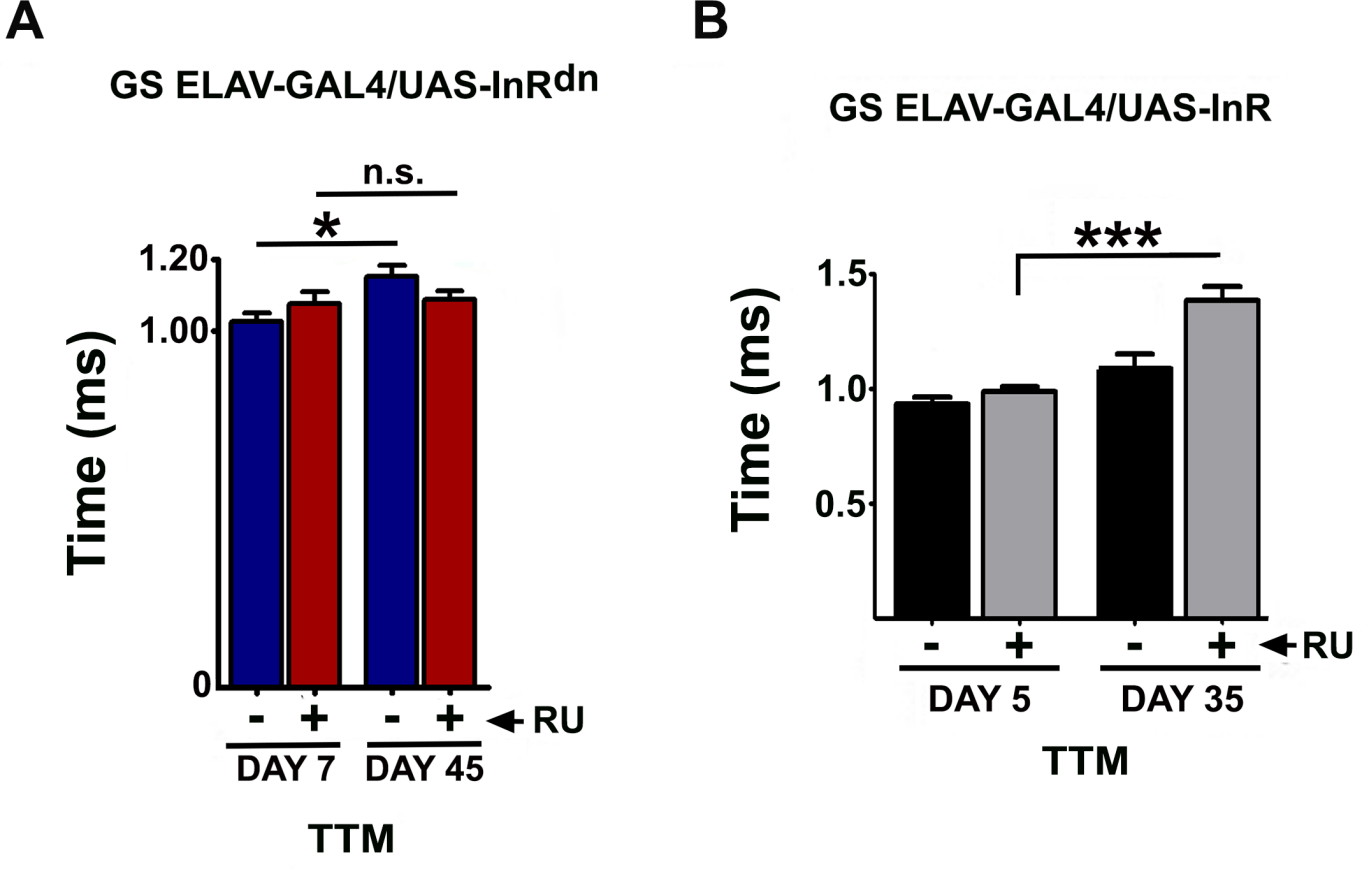
Insulin signaling manipulation in the adult nervous system. (A) Nervous system-specific insulin/insulin-like growth factor signaling (IIS) down-regulation (RU+) prevented age-associated loss of transmission in the tergotrochanteral muscle (TTM) pathway (*n* = 7–10). (B) TTM response latency deteriorated faster with age in flies over-expressing IIS (RU+) (age x treatment interaction: *P* = 0.0105; *n* = 6–9). Both panels: error bars denote SEM.

Constitutive and ubiquitous down-regulation of IIS could affect development of the nervous system and could also act from other tissues, systemically, to maintain GFS function during aging. To address these possibilities, we first confined lowered IIS to adult neurons using the inducible GeneSwitch (*GS ELAV-GAL4*) line [57] to drive expression of the DN IR [58]. Response latency did not differ between induced flies and controls at day 7 and increased with age in control but not induced flies (Fig 3A, S3A Fig). To assess the response to a physiologically relevant stimulus, we activated the GFS using a light air-puff directed to the fly’s head, transmitted to the GFs via mechanosensory afferents [59]. The mean frequency of the DLM responses within 5 seconds was significantly higher in mid-aged RU(+) flies (>70%), demonstrating an enhanced physiological outcome in response to lowered IIS in the nervous system (S3B Fig). Lowered IIS in neurons only during adulthood thus maintained GFS function during aging. If neuronal IIS impairs GFS function during aging, then increased IIS would be predicted to impair it further. Indeed, over-expression of WT IR in adult neurons significantly increased response latencies in mid-aged animals (Fig 3B).

**Fig 4:**
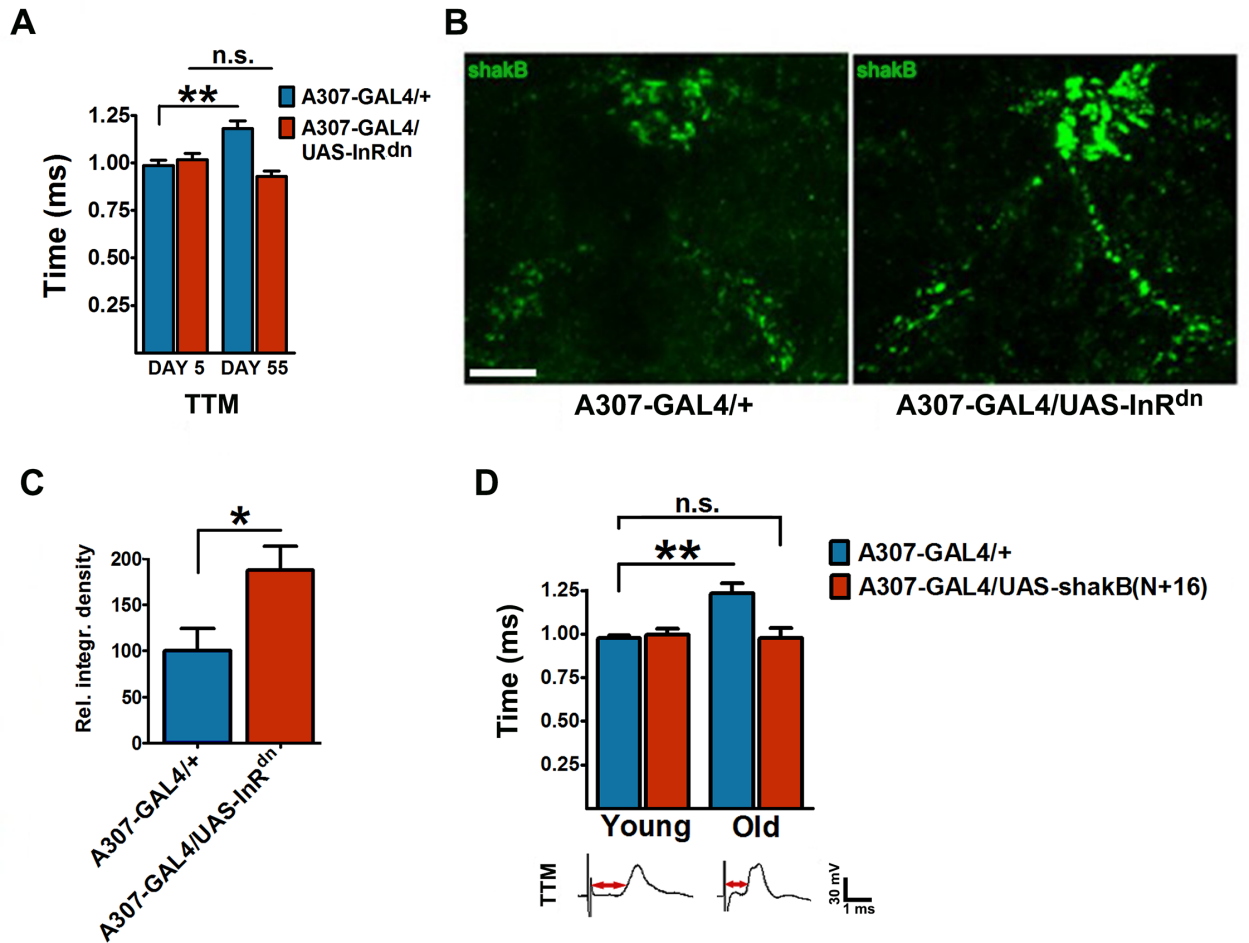
Insulin signaling regulates giant fiber system (GFS) function during aging, and gap junctional density. (A) GFS-specific over-expression of *InR*^*dn*^ abolished the age-related response latency decline (tergotrochanteral muscle [TTM] pathway age x genotype interaction: *P* = 0.0004; *n* = 6–9). (B) Representative images of thoracic shaking-B protein (SHAK-B) staining in 45-day-old control (*A307-GAL4/+*) flies (left), and 45-day-old *A307-GAL4/UAS-InR*^*dn*^ flies (right). Scale bar: 15 μm. (C) Quantification of SHAK-B signal intensities in the bilateral tracts of the GFS (*n* = 4–7). (D) Top: Over-expression of *SHAK-B(N+16)* prevented functional decline in the GFS with age (interaction *P* = 0.015; *n* = 6–14 per genotype/age). Bottom: Representative TTM traces from 45-day-old control (left) and *SHAK-B(n+16)*-over-expressing (right) flies. Red arrows indicate response latency periods. All panels: error bars denote SEM.

To investigate a possible systemic role of lowered IIS in other parts of the nervous system in maintaining GFS function during aging, we drove expression of *InR*^*dn*^ within a small subset of neurons including the GFS itself using the *A307-GAL4 *driver, which drives strongly in the GF interneurons and, to a lesser extent, the TTM and DLM motoneurons and PSI interneurons. This resulted in complete rescue of age-associated increase in response latency (Fig 4A, S4A Fig). We demonstrated the GF neuron-specific effect of lowered insulin signaling by using the recently described *split-GAL4* line [60] that drives expression exclusively in the GF interneurons (S4B Fig).

IIS down-regulation in the GFS also caused a marked increase in the density of thoracic SHAK-B in old flies (Fig 4B and 4C). *SHAK-B* mutants display longer response latencies [54], while forced expression of *SHAK-B(n+16)*, the isoform crucial for functional hemichannel formation in the GFS [61], prevented the age-related functional decline (Fig 4D, S4C Fig) in both branches of the circuit. These results further strengthen the hypothesis that loss of SHAK-B caused the age-associated loss of functionality within the GF circuit. The diameter of GF interneurons in old flies did not vary significantly between the genotypes (S5A Fig), suggesting that the size of principal circuit interneurons cannot account for the measured differences in conduction speed in these animals.

### Lowered insulin signaling stimulates recycling of GJ proteins to the plasma membrane

**Fig 5:**
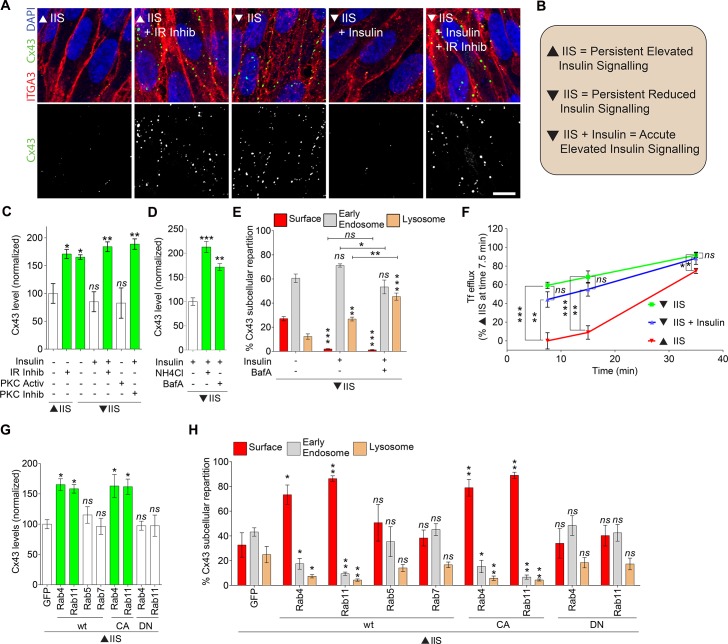
Lowered insulin/insulin-like growth factor signaling (IIS) increases connexin 43 (Cx43) gap junction (GJ) formation in human cells. (A and B) Confocal pictures of human retinal pigment epithelial (RPE1) cell monolayers upon elevated or reduced IIS, stimulated or not with insulin (1 hour) or insulin receptor (IR)/insulin-like growth factor-1 receptor (IGF1R) dual inhibitor (“IR inhib”), as indicated, and immunostained for Cx43 (green), integrin α3 (ITGA3, red), and DNA (DAPI, blue), images are representative of at least 30 captures from 3 independent experiments). Bar, 10 μm. (C, D, G) Quantification from high-throughput microscopy images of the total levels of Cx43 in RPE1 cells upon elevated or reduced IIS, stimulated or not with insulin, IR/IGF1R dual inhibitor (“IR inhib”), protein kinase C (PKC) activator or inhibitor or lysosomal inhibitors (NH_4_Cl or bafilomycin A [BafA]) or transfected with the indicated wild-type (WT), constitutively active (CA) or dominant-negative (DN) Rab constructs, and normalized as indicated. (Data are shown as means ± SEM from 3 independent experiments (over 12,000 Cx43 punctae per condition); *n*.*s*., not significant; *P < 0.05, **P < 0.1, ***P < 0.001; 1-way ANOVA and Dunnett test versus “−insulin,” “+insulin,” or enhanced green fluorescent protein [EGFP], as appropriate). (E and H) Percentage of Cx43 colocalizing with ITGA3 (“Surface”), early endosome antigen 1 (EEA1; “Early Endosomes”) or lysosomal-associated membrane protein 1 (Lamp1; “Lysosomes”) in cells treated as indicated (Data are shown as means ± SEM from 3 independent experiments (over 12,000 Cx43 punctae per condition); *n*.*s*. = not significant; *P < 0.05, **P < 0.1, ***P < 0.001; 1-way ANOVA and Dunnett test versus “−insulin,” “+insulin,” or EGFP, as appropriate). (F) Transferrin efflux (endosomal recycling) measured by flow cytometry from RPE1 cells upon elevated or reduced IIS, stimulated or not with insulin (1 hour), as indicated. (Data are shown as means ± SEM from 3 independent experiments and normalized to “elevated IIS” at 7.5 minutes. Over 10,000 cells were analyzed per condition and per experiment; *n*.*s*., not significant; ***P < 0.001; 1-way ANOVA and Dunnett test.

Because *SHAK-B* transcript levels were unaffected by lowered IIS (S5B Fig), we hypothesized that SHAK-B protein level was regulated by degradation, but saw no impact of lowered IIS on age-related loss of proteasomal proteolytic activity (S5C Fig). We therefore investigated the effect of reduced IIS on trafficking into lysosomes. To test this, we initially used confluent human retinal pigment epithelial (RPE1) cells, because the formation and trafficking of GJs can be studied in high temporal and spatial resolution and can be easily manipulated in this system. With persistently elevated IIS (the cells were maintained in insulin-supplemented complete medium throughout the experiments; S6A Fig), the cell surface levels of the main GJ protein connexin 43 (Cx43), but not that of the glucose transporter 1 (GLUT1) or integrin α3 (ITGA3), was low (Fig 5A, S6A–S6E Fig), consistent with the short half-life and rapid turnover of GJs in tissue culture models [62]. Lowering IIS by incubating cells in serum-free media containing glucose but no insulin for 13 hours (S6A Fig) or by using a dual inhibitor for the IR and IGF-1 receptor (IGF-1R) for 1 hour, increased the levels of GJs to 165 +/− 4.20 and to 171 +/− 8.13% (*P* < 0.05, *P* < 0.01), respectively (Fig 5A–5C). Upon reduced IIS, an acute (1 hour) stimulation with insulin (S6A Fig) was sufficient to decrease the levels of Cx43 down to that of cells subjected to constant elevated IIS or to acute activation of protein kinase C (PKC), a known mediator of Cx43 internalization [62] (Fig 5A–5C). Cx43 accumulated at the cell surface (labeled by ITGA3) under decreased IIS, but not upon acute insulin stimulation in which it was significantly shifted into lysosomal-associated membrane protein 1 (LAMP1)-positive lysosomes (Fig 5E). These phenotypes could have been achieved by a change in either endocytosis or endosomal recycling. Acute insulin stimulation did not change endocytosis and endosomal recycling of the transferrin receptor—which cycles constitutively between endosomes and the plasma membrane (Fig 5F, S6F Fig). However, elevating IIS induced targeting of Cx43 to lysosomes and degradation, which could be blocked upon inhibition of lysosomal activity by NH_4_Cl or bafilomycin A (BafA) treatments (Fig 5D and 5E) or reverted by over-expressing the small GTPases Rab4 or Rab11 (Fig 5G and 5H), which regulate recycling to the plasma membrane of synaptic receptors, gap junctional proteins, and ion channels in *Drosophila* [63–65] and mammals [66,67].

Over-expressing Rab5 or Rab7 (which regulate early endosomes or late endosomes-lysosomes formation, respectively) could not revert the phenotypes (Fig 5G and 5H). Consistent with these findings, over-expression of the constitutively active (CA) forms of Rab4 or Rab11, but not that of their DN forms, derouted Cx43 from the degradative to the recycling pathway and blocked degradation (Fig 5G and 5H), thus mimicking lowered IIS. Inhibiting lysosomal functions was sufficient to revert Cx43 levels but not plasma membrane localization upon elevated IIS (Fig 5D and 5E), further confirming that elevated IIS targets GJ proteins to lysosomes for their degradation. Cumulatively, these results show that reducing IIS enhanced the formation of GJs in human cells by stimulating their recycling to the plasma membrane, and decreasing their trafficking into lysosomes and subsequent degradation, consistent with the higher levels of SHAK-B protein in the IIS mutant flies.

### Recycling-mediating Rabs rescue age-associated decline of GFS function and reduction in gap junctional density

**Fig 6:**
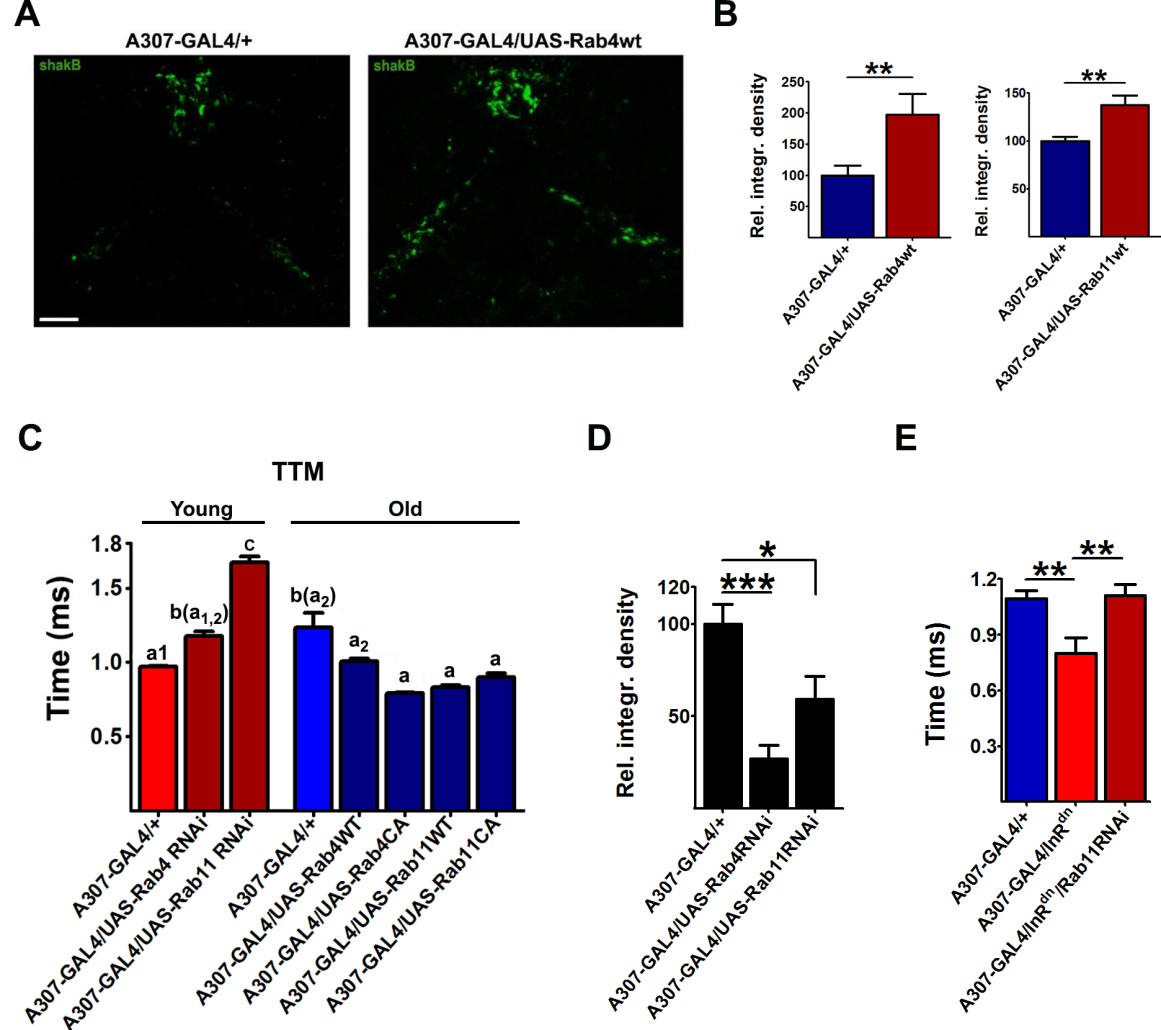
Over-expression of recycling Rabs rescues age-related loss of gap junctions (GJs) and giant fiber system (GFS) function. (A and B) Over-expression of Rab4 (wild type [WT]) or Rab11(WT) in the GFS led to increased levels of shaking-B protein (SHAK-B) in the thorax of old flies. Representative confocal images for Rab4(WT) are shown in (A), the quantification in (B) (*n* = 5–10). (C) Tergotrochanteral muscle pathway (TTM) response latencies from young ([y] days 5–7) and old ([o] days 45–50) flies of various genotypes. WT and constitutively active (CA) construct over-expressed WT or CA forms of Rab4 and Rab11, respectively. Bars with different first letters indicate significant difference (irrespective of the subscript). The letters in the parentheses indicate a lack of significance with the specified bar (*n* = 4–8). (D) Quantification of the SHAK-B signal intensity in the bilateral tracts of young (7-day-old) flies with silenced Rab4 or Rab11 expression (*n* = 8–10). (E) Rab11 is indispensable for the effect of reduced signaling on the transmission through the TTM branch of the GF circuit (*n* = 5–13). Error bars denote SEM.

Consistent with our in vitro results, over-expression of Rab4 or Rab11 in the *Drosophila* GFS led to increased SHAK-B density in the thorax of 40- to 45-day-old animals (Fig 6A and 6B). Furthermore, overexpression of WT or CA Rab4 or Rab11 completely blocked the age-related increase in response latency (Fig 6C, S7A Fig). Down-regulation of Rab4 and Rab11 by RNAi, on the other hand, increased response latencies even in young flies (Fig 6C, S7A Fig), consistent with the effect of Rab4 and Rab11 suppression on thoracic SHAK-B density in young adults (Fig 6D, S7B Fig). Thus, experimentally increasing the expression of these Rabs could rescue loss of GJs and of GFS function during aging, and their suppression could impair neurotransmission in young flies by reducing synaptic SHAK-B levels. Furthermore, Rab11 was required for reduced IIS to maintain response latency in old flies (Fig 6E, S7C Fig), supporting the idea that reduced IIS exerted its synaptic effects through the recycling-mediating Rabs.

## Discussion

A number of experimental results demonstrated the impact of long-term IIS manipulations on the nervous system. For example, systemic injections of IGF-1 mimicked some of the effects of exercise in the brain [68], and genetically reduced IGF-1 signaling in the whole organism reduced inflammation and neuronal loss in a mouse Alzheimer disease model [32]. Likewise, chronic IIS manipulations only in the nervous system can have consequences on the whole organism: attenuated IR substrate/IR substrate 2 signaling in aging brains promoted healthy metabolism and extended the lifespan in mice [69], and neuron-specific reduction of IIS increased longevity in *Drosophila* [70]. At the synaptic level, basal IGF-1 activity has recently been shown to regulate ongoing neuronal activity in hippocampal circuits [26]. While infusion of IGF-1 does not appear to have short-term influence on Cx43 levels in various regions of the rat brain [71], no study so far has examined the effect of chronic IIS manipulations in the aging nervous system on GJs.

**Fig 7:**
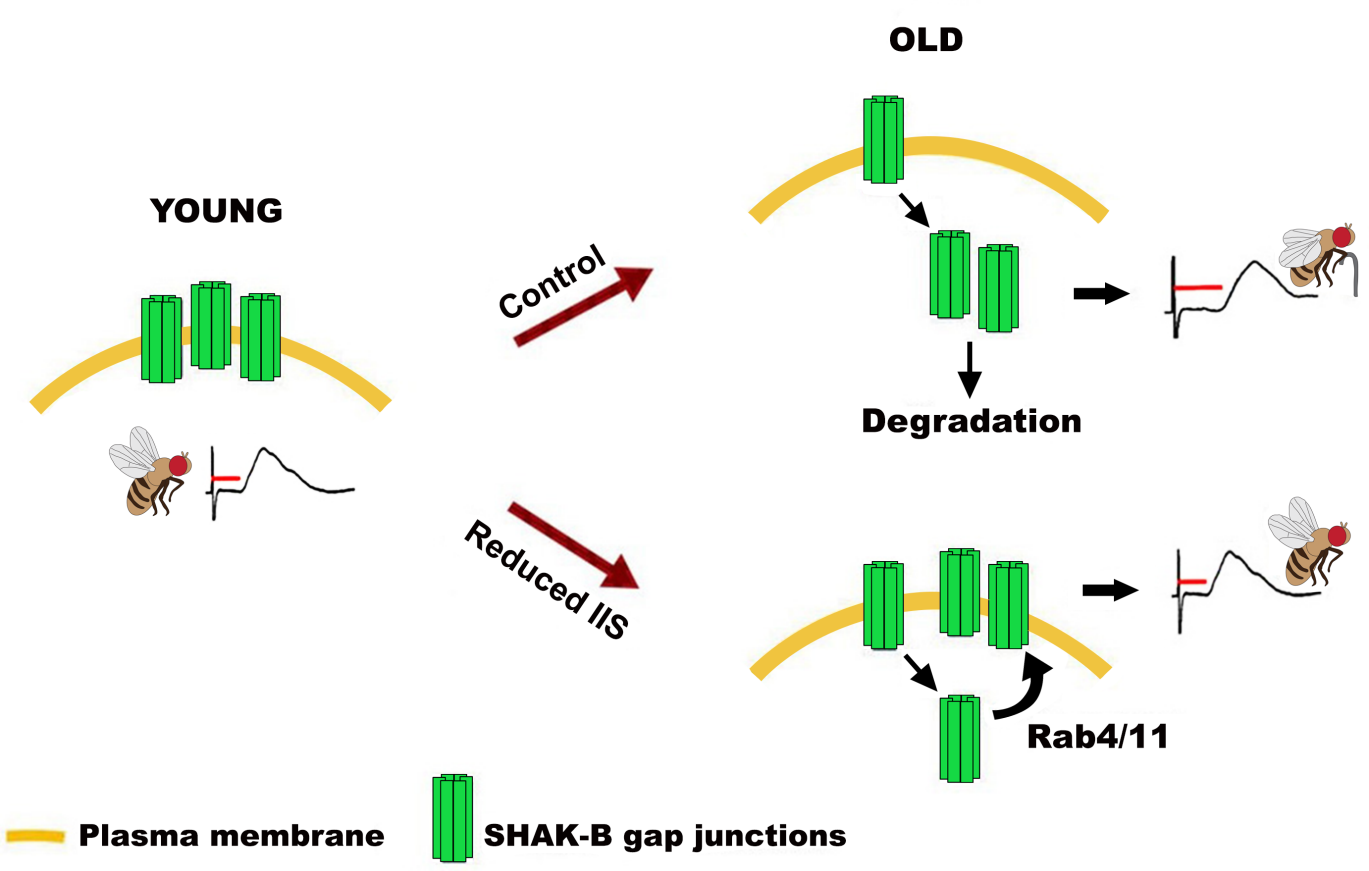
Model for the age-related effect of reduced insulin/insulin-like growth factor signaling (IIS) on gap junctional density and electrophysiological properties of the giant fiber system (GFS). Attenuated insulin signaling prevents the loss of shaking-B protein (SHAK-B)-containing gap junctions (GJs) and response latency increase with age by stimulating plasma membrane recycling of GJ subunits via Rab4 and/or Rab11.

In this work, we demonstrated a role for IIS in regulating the trafficking of gap junctional proteins that is conserved over the large evolutionary distance between *Drosophila* and humans, and between different cell types. Elevated IIS induces the targeting of GJ proteins to lysosomes and degradation, thereby decreasing their cell surface assembly (Fig 7).

Specifically, reduced insulin signaling throughout adulthood leads to Rab4/11-mediated increase in the synaptic targeting of *SHAK-B*-encoded gap junctional components in the *Drosophila* escape response circuit, resulting in the maintenance of the “youthful” functional output even in old flies. Previous studies demonstrated a positive effect of reduced insulin signaling on neuronal circuit function. For example, visual acuity in is improved in mice with reduced insulin signaling in the visual cortex [72]. In the nematode *C*. *elegans*, mutations of the IR gene resulted in improved chemical transmission at the neuromuscular synapse, and delayed decline in the synaptic function with age [73]. Our findings have revealed a novel restorative and adaptive cellular mechanism by which lowered IIS can maintain electrical transmission in a neuronal circuit during aging, and that could potentially be harnessed to prevent decline in neuronal function. A recent report [70] demonstrated a negative effect of neuron-specific IIS reduction on age-specific walking behavior in *Drosophila*, suggesting that the effect of insulin signaling depends on the type of neuron(s) mediating a specific behavior. For example, physiological roles of different (chemical) neuronal circuits can be preferentially mediated by either evoked or spontaneous transmission [74]. Interestingly, blockade of insulin signaling has opposing effects on these 2 types of transmission [26], possibly explaining some of the seemingly contradictory experimental data about the role of IIS in the nervous system. Together, these findings indicate that studies of insulin signaling in the nervous system should be circuit- and synapse type-specific, taking into consideration the physiological properties of the neuronal system under study, and precluding simplified generalizations about the effectiveness of specific IIS manipulations across the nervous system.

## Materials and methods

### Fly stocks and husbandry

Ubiquitous and neuron-specific expression was achieved with the *GAL4-*dependent upstream activator sequence (*GAL4-UAS*) system [50]. *Daughterless(da)-GAL4* flies (*w1118; P*^*1*^*3* [#8641]) were obtained from the Bloomington *Drosophila* Stock Center (BDSC); *GS ELAV-GAL4* was derived from the original *GS ELAV 301*.*2* line [57] and obtained as a generous gift from Dr. H. Tricoire (CNRS, Paris, France). The *UAS-InR*^*dn*^ (BDSC #8252) transgene encodes an amino acid substitution in the kinase domain (K1409A) of the *Drosophila* IR (dInR), resulting in its DN activity [75]. The *A307-GAL4* and *UAS-SHAK-B(N+16)* lines were a kind gift from Dr. P. Phelan (University of Kent, Canterbury, UK); the *UAS-Rpn11* line was a gift from the lab of Dr. M. Miura (University of Tokyo, Tokyo, Japan), the *split-GAL4* was a gift from G. Card (Janelia Farm, Ashburn, VA); these stock are a combination of 2 *split-GAL4* halves; 1 has the activation domain of the *GAL4* and the other has the DNA binding domain. Only the cells that express both halves will reconstitute a complete *GAL4* [76]. Other BDSC stocks include the following: *UAS-Rab4(WT)* (#23269), *UAS-Rab4(CA)* (#23268), *UAS-Rab4(RNAi)* (#33757, TRiP), *UAS-Rab11(WT)* (~8506), *UAS-Rab11(CA)* (#9791), *UAS-Rab11(RNAi)* (#42709) and *UAS-InR* (#8262). To standardize genetic background, parental *GAL4* and *UAS* strains used to generate experimental and control genotypes were back-crossed to laboratory control strain *w*^*Dah*^ (Wolbachia-infected) for at least 6 generations, beginning with an initial cross between w^Dah^ females and transgenic males, followed by 5 subsequent back-crosses between transgenic females and *w*^*Dah*^ males. The white Dahomey (w^Dah^) stock was derived by incorporation of the *w*^*1118*^ mutation into the outbred Dahomey background by back-crossing. All stocks were maintained and all experiments were conducted at 25°C on a 12 hour to 12 hour light:dark cycle at constant humidity using standard sugar/yeast/agar (SYA) media (15gl^−1^ agar, 50 gl^−1^ sugar, 100 gl^−1^ autolyzed yeast, 100gl^−1^ nipagin, and 3ml l^−1^ propionic acid) [77]. In RU experiments, adult-onset neuronal expression was induced by adding mifepristone (RU486; Sigma -Aldrich, St. Louis, MO) to the standard SYA medium at 200 mM starting at day 1 post-eclosion. For all experiments, flies were reared at standard larval density and eclosing adults were collected over a 12-hour period. Flies were mated for 48 hours before separating females from males. Female flies were used in all experiments.

### Electrophysiology

Recordings from the GFS of adult flies were performed as described by Allen et al. [78]; a method based on those described previously [44,79]. Flies were anaesthetized by cooling on ice and secured in wax placed inside a small Petri dish, ventral side down, with the wings held outwards in the wax to expose lateral and dorsal surfaces of the thorax. A tungsten earth wire served as a ground electrode and was placed in the abdominal cavity. Extracellular stimulation of the GF neurons was achieved by placing 2 electrolytically (NaOH) sharpened tungsten electrodes through the eyes and into the brain (the supra-oesophageal ganglion) to deliver a 40V pulse for 0.03 ms using a Grass S48 stimulator. Threshold for the short-latency, direct excitation for GF stimulation was previously demonstrated to be a 10 to 20 V pulse that lasts 0.03 ms [44,48]. We therefore applied pulses 2 to 3 times threshold to ensure that threshold was always exceeded.

Intracellular recordings were made following GF stimulation from the TTM and contralateral DLM muscle using glass micropipettes (resistance: 40–60 MΩ). The possibility that descending neurons other than the GFs might be simultaneously activated, leading to a possible TTM or DLM response, was previously excluded [78]. The electrodes were filled with 3M KCl and placed into the muscle fibers through the cuticle. Responses were amplified using Getting 5A amplifiers (Getting Instruments, San Diego, CA) and the data digitized using analogue-digital Digidata 1320 and Axoscope 9.0 software (Molecular Devices, Sunnyvale, CA). For response latency recordings, at least 5 single stimuli were given with a 5-second rest period between each stimulus; measurements were taken from the beginning of the stimulation artifact to the beginning of the EPSP (i.e., muscle depolarization). For direct activation of motoneurons (“thoracic stimulation”) [42], stimulating electrodes were removed from the brain and placed at the anterior end of the thorax through the cuticle and into the fused ganglia in the ventral region of the thorax. The signals were amplified and stored on a PC with pCLAMP software and a DMA interface board (Molecular Devices). Analysis was performed on the PC using pCLAMP and Microsoft Excel 2010 software (Microsoft, Seattle, WA).

### *Drosophila* immunocytochemistry and microscopy

Nervous systems (for the SHAK-B staining) were dissected in *Drosophila* saline [S3], fixed in 4% para-formaldehyde in phosphate buffered saline (PBS) for 30 minutes at room temperature and washed in PBS. After pre-incubation in blocking solution containing 4% normal goat serum in PBS + 0.5% Triton X-100 (PBT-X), preparations were incubated overnight at 4°C in primary antibodies diluted in blocking solution. Primary rabbit anti-SHAK-B antibody, raised against a C-terminal peptide common to all members of SHAK-B group of proteins (gift from P. Phelan), was used at 1:100. Preparations were washed 5 times in PBT-X and incubated with Alexa Fluor488-conjugated goat anti-rabbit secondary antibodies (Molecular Probes, 1:500) for 2 hours. Images were taken on Leica TCS SP2 inverted confocal microscope (Leica Microsystems GmbH, Wetzlar, Germany) or Zeiss 700 (Carl Zeiss MicroImaging GmbH, Jena, Germany). Three- to 5-day-old flies were used for immunostaining with anti-InR antibody (Cell signalling, #3024) at a concentration of 1:1000 followed by goat anti-rabbit dylight 649 secondary antibody at a 1:1000 concentration. In most cases, CNS preparations from animals with different genotypes were mounted on the same slide to control for possible variability in mounting procedure or properties of cover slips. For all preparations on the slide the same confocal settings were used (zoom, laser strength, PMT, gain, digital offset, averaging), and images represent projections of confocal z series composed of approximately 20 focal planes (slices) taken at approximately 1 μm steps at 400x magnification. Images were generated using the sum slices option in ImageJ (NIH, Bethesda, MD) that creates a real image that is the sum of pixel intensities in all focal planes. The abundance of GJs (labelled by anti-SHAK-B antibody) in the SHAK-B-positive bilateral tracts in the mesothoracic neuromere (T2) was quantified by drawing a line around the SHAK-B signal around each SHAK-B-labelled tract, and measuring the area and mean grey value (MGV). The area and MGV are first calculated for each tract separately; weighted mean MGV was then calculated for both tracts together and integrated density obtained as mentioned above.

### GF dye injections and diameter measurements

The GF axons were injected in the connective with a dye solution of 10% w/v Neurobiotin (Vector Laboratories, Burlingame, CA) and tetramethyl rhodamine-labeled dextran (Invitrogen, Carlsbad, CA) in 2 M potassium acetate by passing depolarizing current, respectively. For GJ labelling, rabbit anti-SHAK-B (1:100) and goat anti-rabbit Dylight 649 (1:1000; Jackson ImmunoResearch Laboratories, West Grove, PA) was used. Confocal images were obtained using a Nikon A1 plus confocal with an Apo 60X oil lambdaS objective. Axon diameters of rhodamine-dextran and neurobiotin labelled GFs were measured in the first thoracic ganglion. Nikon Elements Advanced Research 4.4 Binary Editor was used to trace and 3D reconstruct the GF and ND Images Arithmetic function was used to extract anti-SHAK-B labelling that localizes to the 3D reconstructed GFs.

### Western blots

All western blots were run on 10% nongradient sodium dodecyl sulphate poly-acrylamide gel and proteins were transferred onto nitrocellulose membrane using a semi-dry blotter (Bio-rad). For ChAT (choline acetyltransferase) western blots, 10 CNS were dissected in PBS, boiled twice in Laemmli sample buffer and run on a 10% nongradient sodium dodecyl sulphate (SDS) polyacrylamide gel. Proteins were transferred onto nitrocellulose using a semidry blotter (Bio-rad) and probed with mouse monoclonal anti-ChAT 4B1 antibody (Developmental Studies Hybridoma Bank, Iowa City, Iowa) at [1:100] in 5% milk + TBST. Detection was performed with anti-mouse horseradish peroxidase-conjugated secondary antibody and Amersham ECL detection reagent (GE Healthcare, Little Chalfont, UK). Bands were normalized to actin.

Western blots for the recycling of membrane components and for Dα7 (nAChR) used fly heads, homogenized and boiled in Laemmli sample buffer (10μL/lane). Western blots were run as above. Primary *Drosophila* anti-Dα7 antibody concentration (incubation was performed overnight at 4°C) was 1:1000 [56]; the incubations were done in 3% BSA/TBS-T. Bands were normalized to actin, using (Abcam, Cambridge, UK) mouse anti-Actin [1:10000] in 5% milk/TBS-T. Secondary antibodies were diluted [1:10000] in 3% BSA/TBS-T, using either (Abcam) goat anti-mouse HRP (Abcam), or goat anti-rabbit HRP or anti-rat HRP (Sigma-Aldrich). Detection was performed with Luminata^TM^ Crescendo or Forte (for Dα7) western blot HRP substrate (#WBLUR0500; Millipore, Billerica, MA) and imaged using Image Quant LAS4000.

### Quantitative RT-PCR

RNA was extracted from 8 CNS, dissected in PBS, with 2 x 500 ul of TRIzol (Invitrogen) using a RiboLyser homogenizer and precipitated overnight at −20°C using 1 volume of isopropanol. Glycogen (Invitrogen) was included at 50 μg/ml to act as a carrier. The pellet was washed in 2 x 1 ml of 75% ethanol and re-suspended in DEPC water. RNA was treated with TURBO DNase (Ambion; Thermo Fisher Scientific, Waltham, MA) and reverse transcribed using Superscript II (Invitrogen) and Oligo dT. SHAK-B RNA was quantified with real-time PCR using primers (forward: (CAACGCACAACCAAAAAGG, reverse: GCGAAAAACAGGTGAATCG). Total RNA levels were compared after normalization to tubulin. The gene of choice for normalization was selected following a comparison of its expression level with actin and Tat using NormFinder.

### Proteasome activity assay

Fly heads were homogenized in 25mM Tris, pH 7.5, and protein content determined by Bradford assay. Chymotrypsin-like peptidase activity of the proteasome was assayed using the fluorogenic peptide substrate Succinyl-Leu-Leu-Val-Tyr-amidomethylcoumarin (LLVY-AMC), based on a previously published protocol [80]. Twenty micrograms of crude fly head homogenate total protein was incubated at 37°C with 25 μM LLVY-AMC in a final volume of 200 μLs. Enzymatic kinetics were conducted in a temperature-controlled microplate fluorimeter (Infinite M200; Tecan, Männedorf, Switzerland), at excitation/emission wavelengths of 360/460 nm, measuring fluorescence every 2 minutes for 30 minutes. Proteasome activity was determined as the slope of AMC accumulation over time.

### Plasmids, reagents, and antibodies

Human Rab4a, Rab4a N121I (DN mutant, called “Rab4 DN” in this study), Rab4a Q70L (CA mutant, called “Rab4 CA” in this study), Rab5a, Rab7a, Rab11a, Rab11a S25N (DN mutant, called “Rab11 DN” in this study), and Rab11a Q70L (CA mutant, called “Rab11 CA” in this study) genes were cloned into pEGFP-expressing vectors. Antibodies used for immunofluorescence analysis were rabbit polyclonal anti-Cx43 (called “Cx43” in this study; 3512; Cell Signaling Technologies, Danvers, MA); mouse monoclonal anti-integrin α-3 (called “ITGA” in this study; GTX11767; Genetex, Irvine, CA); rabbit polyclonal anti-GLUT1 (15309; Abcam); rabbit anti-TfR antibody (CBL47; Millipore); mouse anti-EEA1 (MAB8047; Bio-techne, Minneapolis, MN), mouse anti-LAMP1 (DSHB H4A3), and mouse and rabbit IgG isotype controls (DA1E and G3A1; Cell Signaling). Alexa Fluor 488-, 555-, and 633-conjugated donkey, anti-mouse, and anti-rabbit antibodies were from Life Technologies (Durham, NC).

Insulin (used at 1 μM for 1 hour, unless indicated [MP Biomedicals, Santa Ana, CA]), BMS 536924 (called IR inhib in this study and used at 5 μM [4774; Tocris, Bristol, UK]), phorbol 12-myristate 13-acetate (PMA; called PKC active in this study and used at 100 nM for 1 hour [10008014; Cayman Chemical, Ann Arbor, MI]), Gö6983 (called PKC inhib in this study and used at 5 μM for 1 hour [2285; Tocris]), NH_4_Cl (Sigma-Aldrich), BafA 1 (called BafA in this study and used at 1 μM [J61835; Alfa Aesar, Haverhill, MA), DAPI (Sigma-Aldrich), Alexa Fluor 488-conjugated transferrin (referred to as Tf,-A488 and used at 200 μg/ml [Life Technologies]), desferroxamine (Sigma-Aldrich), and holo-transferrin (Sigma-Aldrich).

### Cell culture and IIS manipulations

RPE1 cells (ATCC CRL-4000) were maintained at 37°C, 5% CO_2_, in complete medium (DMEM:F12 HAM (1:1 v/v [D6421; Sigma-Aldrich]) supplemented with 10% fetal bovine serum (FBS [Life Technologies), 0.5% (w/v) sodium bicarbonate [Sigma-Aldrich], 2 mM GlutaMAX [Life Technologies], antibiotic-antimycotic [Sigma-Aldrich], and 20 μg/ml hygromycin). Cells were regularly tested for mycoplasma contamination. For all assays RPE1 cells were seeded in appropriate culture dishes (approximately 8 x 10^4^, 2 x 10^5^; cells were seeded in each well in a 96-well glass bottomed or 12-well plastic tissue culture dish, respectively) and grown as monolayers for 4 days in complete medium (containing insulin from FBS). Persistent elevated IIS (elevated IIS) was achieved by maintaining the cells thorough the experiment in complete medium. Persistent reduced IIS (reduced IIS) was achieved by incubating the cells in serum-free medium (containing glucose but no insulin) for 13 hours. Alternatively, cells were incubated in insulin-free medium (DMEM:F12 HAM (1:1 v/v) supplemented with 7.5 mM GlutaMAX (Life Technologies), antibiotic-antimycotic (Sigma-Aldrich), 1 μg/mL hydrocortisone (Sigma-Aldrich), 50 μg/mL ascorbic acid (Sigma-Aldrich), and 5 ng/mL basic FGF (Life Technologies) for 13 hours. IIS was acutely reduced by treating cells maintained in complete medium with IR and IGF-1R dual inhibitor for 1h (elevated IIS + IR inhib). Acute IIS (reduced IIS + insulin) was achieved by incubating the cells in serum-free medium (containing glucose but no insulin) for 12 hours, followed by addition of insulin (10 nM to 1 μM for 1 hour). In some experiments, cells grown under elevated IIS or reduced IIS conditions were treated with PKC activator (PMA, 100nM), PKC inhibitor (Gö6983, 5 μM), NH_4_Cl (50 mM) or BafA 1 (1 μM) with or without insulin for 1 hour.

### DNA transfection

All transfections were undertaken using GeneJuice (Millipore) transfection reagent according to the manufactures instructions in 96-well glass bottomed dishes at 100 ng of DNA per 96 well. Following 36 hours of transfection, cells were treated for experimentation and fixed, then prepared for analysis.

### Fixation and fluorescence labeling for microscopy

Treated cell monolayers were fixed in 4% paraformaldehyde (PFA) in PBS for 20 minutes at room temperature, followed by 2 washes with PBS and then incubated for 1 hour in 50 mM NH_4_Cl in PBS to quench the residual PFA. Cells were then surface-labeled with anti-ITGA3 followed by permeabilizaton with 0.1% Saponin-PBS and immuno-labeled in the presence of horse serum (Life Technologies) at an antibody dilution of 1:200 for all other primary antibodies, 1:1000 for all secondary antibodies, and 1:10,000 for DAPI staining of DNA. Fluorescently-labeled samples were imaged using either a high-throughput automated epifluorescence microscope (ImageXpress Micro XLS; Molecular Devices) or a confocal laser-scanning microscope (LSM700; Carl Zeiss MicroImaging).

### Image analysis

Image analysis was performed using a protocol established in CellProfiler image analysis software [81]. A set of image analysis algorithms, or pipeline, was constructed to measure the properties of interest within the RPE1 cell culture labelled with DAPI, anti-Cx43 and/or anti-ITGA3, anti-EEA1, and anti-Lamp1, as required. Each image-set, corresponding to 1 field of view or site and comprising 3, or 4 where required, fluorescent channels, were analyzed independently using this pipeline. Twelve sites per well were analyzed and repeated in triplicate experiments.

In brief, an illumination correction function was calculated for each channel using a median filter (200 x 200 pixels) to correct for illumination variations across each 96-well plate. Each image set was then processed in an imaging pipeline as follows. The 4 channels' raw images were divided by their respective plate/channel illumination function. Firstly, segmentation of the nuclei of each cell in the field of view was identified corresponding to an arbitrary fluorescence intensity, median size (20 to 60 pixels) and shape (circular) according their DAPI DNA labelling. Next, cells were identified extending from the nuclei using an arbitrary fluorescence intensity corresponding to the red fluorescent channel. Identified cells were further partitioned, where appropriate, into transfected or nontransfected populations by their fluorescence associated with their GFP expression. Next, punctae corresponding to Cx43 and either LAMP1 or EEA1 were identified within the masked cellular region, with a typical diameter range of range of 8 to 20 pixels and having an arbitrary threshold of fluorescence intensity associated with their corresponding antibody labelling were identified as primary punctae objects for analysis. Finally, where appropriate, the surface of the cells was identified by labelling associated with anti-ITGA3 in nonpermeabilized samples, which were subsequently permeabilized and labelled with DAPI and Cx43. This ITGA3 labelling was designated as an object surface. The intensity, frequency, and size of all punctae were measured, and the ITGA3 labelled surface area was also measured. Cx43 punctae intersecting with early-endosomes and late-endosomes synapses were subsequently identified if 2 primary punctae objects in the case of early (Cx43/EEA1) or late (Cx43/LAMP1) were determined to colocalize in their respective fluorescent channels. The intensity and frequency for the colocalized object were measure for all channels. Cx43 objects localized to the surface were identified if Cx43 designated primary punctae objects intersected with ITGA3 labelled surface objects. All subsequent object data were partitioned into either transfected or nontransfected groups where appropriate. Levels of cellular Cx43 data were calculated using numbers of punctae per field of view normalized to cell number, and the intensity of the identified punctae. Levels of Cx43 localized to surface were normalized total number of Cx43 and their intensity, to surface area and the cell number in each of view. Levels of Cx43 localized to early-endosomes and late-endosomes were normalized to the total number of Cx43, and their intensity and the cell number in each field of view and expressed as a percentage of this value. Fractional results of the 3 subcellular compartments measured are expressed as a percentage of their values normalized to a total of 100%.

Data per well were determined by first aggregating the data of images taken within the same well for all sites and then over replicate wells and experiments. The average number of RPE1 cells per condition per experiment were >1,000. The median numbers of Cx43 punctae, EEA1 punctae, or LAMP1 punctae measured per condition per experiment were >12,000, >8,000, and >30,000, respectively.

### Quantification of GJ Cx43 plaques

Treated samples were then fixed and immunolabeled as outlined above. Epi-fluorescent, three-dimensionalprojections were created for each sample by acquiring a z-stack of images using a slice increment of 0.4 μm with 63 x oil objective. A three-dimensional reconstruction of the field of view was created using the image slices and Volocity image analysis software. Individual Cx43 GJ plaques from cells were selected automatically using a protocol established in Volocity image analysis software (Perkin Elmer, Waltham, MA). GJ plaques were identified as objects within a volume range of 5 to 100 μm^3^, according to an arbitrary threshold of fluorescence intensity associated with anti-Cx43 antibody labeling. Once individual objects (GJ plaques) were identified, their mean fluorescent signal and frequency of occurrence were quantified using Volocity software. These values were further partitioned, where appropriate, into those arising from transfected and non-transfected cells, determined by GFP. At least 10 fields of view from each condition were quantified for each sample with approximately 400 plaques being detected per field of view. Where possible, anti-ITGA3 or anti-Glut1 labeling determined the cell–cell interface and were utilized to assess Cx43, ITGA3, or Glut1 cell–cell interface localization through pixel–pixel colabeling.

### Fixation and fluorescence labeling for flow cytometry

Treated cell monolayers were detached using enzyme free cell-dissociation solution and resuspended in ice-cold PBS. Cells were further washed twice in ice cold PBS, and once in acid wash solution (150 mM NaCl, 100 mM Glycine, 5 mM Kcl, 1 mM CaCl_2_, pH4.5), as required. Cells were then fixed in 4% PFA on ice for 5 minutes followed by 20 minutes at room temperature. The fixation reaction was then quenched for 1 hour in the presence of 50 mM NH_4_Cl in PBS. Cells were washed twice in PBS and then immunolabeled as required with appropriate primary antibodies at a dilution of 1:200 in the presence of horse serum and 0.1% saponin in PBS (total staining) or just PBS (surface staining), as appropriate. Control cells were left unlabeled or labeled with an isotype control. This was followed by secondary antibody labeling with anti-rabbit Alexa Fluor 488 at a dilution of 1:1000. A total of over 10,000 events were analyzed per condition on a FACS LSRII flow cytometer (Becton Dickinson) for fluorescence intensities associated with Alexa fluor 488 labeling. Unlabeled and isotype control labeled cells were used to calibrate the instrument settings and to determine background fluorescence labeling respectively. Data were analyzed with FlowJo 8.6.3 (Tree Star Inc., Ashland, OR) software. Results were determined using fluorescence associated with the 488 nm laser excitation and resultant emission per cell and represent the geometric mean of each population.

### Tf recycling assay

Treated cell monolayers were incubated with 200 μg/ml Alexa Fluor 488-conjugated Tf at 37°C for up to 45 minutes (to fully saturate the endocytic network) in appropriate media supplemented with indicated reagents. Internalization was halted by chilling on ice, and cells were washed 3 times with ice-cold PBS to remove unbound Tf and once with mild acid wash buffer (150 mM NaCl, 100 mM Glycine, 5 mM KCl, 1 mM CaCl_2_, pH4.5) to remove surface-bound Tf. Cells were then incubated with prewarmed media (associated with the relevant conditions) containing 0.1 mM desferroxamine and 0.5 mg/ml unlabeled holo-Tf (Sigma-Aldrich) at 37°C for up to 30 minutes. At the indicated timepoints, cells were then incubated on ice, washed with ice-cold PBS, collected, fixed and processed for flow cytometry analysis. Fluorescent Tf remaining in cells at each time point were measured and the efflux rate was expressed as percentage of initial intracellular Tf measurement of resting cells after 7.5 minutes of efflux. Results were further normalised against the saturated level of intracellular Trf488 at time 0 after 45 minutes pre-incubation uptake. Results displayed comprise the fluorescence associated with Trf488 labeling per cell normalized as outlined and represent the geometric mean of each population.

### Surface and total levels of transferrin receptor

Treated cell monolayers were collected using enzyme free cell dissociation solution, fixed and either immunolabelled in the presence of 0.1% Saponin-PBS (for total transferrin receptor [TfR]) or with PBS (for surface TfR) with anti-TfR antibody at a dilution of 1:200 at RT, or with isotype control under the same conditions. All samples were then secondary antibody labeled with Alexa fluor 488 and processed for surface and total TfR quantitation by flow cytometry. Results were determined from the ratio of surface TfR to total TfR using the geometric mean of each population. This value was used to normalize the results from associated samples in the Tf uptake assays.

### Tf internalization assay

Cells were prepared for the assay in the same way as above (surface and total TfR) and then incubated with 200 μg/ml Alexa Fluor 488-conjugated Tf at 37°C for up to 10 minutes in appropriate media supplemented with the indicated reagents. Internalization of Tf was halted by putting the cells on ice and washing immediately with ice-cold PBS. Surface bound Tf was further removed by mild acid washing followed by 3 ice-cold PBS washes. Cells were then detached with enzyme-free cell dissociation solution, fixed and processed for flow cytometry as outlined above. Results were normalized against the ratio of surface TfR to total TfR and control cells and represent the geometric mean of each population.

### Statistical analyses

Statistical analyses were performed using GraphPad Prism 5 software (GraphPad Software Inc., La Jolla, CA). A 2-way ANOVA test was used to perform (age x genotype) interaction calculations. For other comparisons between 2 or more groups, a 1-way ANOVA followed by a Tukey-Kramer post hoc test was used. In all instances, *P* < 0.05 is considered to be statistically significant (*P < 0.05; **P < 0.01; ***P < 0.001). Log-rank tests were performed for survival. All data were tested for Gaussian distribution with Kolmogorov-Smirnov test with the Dallal-Wilkinson-Lillie for corrected *P* value. In case of Gaussian distribution, the following parametric tests were used: Student *t* test (2 groups) or 1-way ANOVA and Dunnett test (2+ groups), as appropriate. All values are reported as the mean ± SEM.

## Supporting information

S1 Fig Response latencies through the DLM pathway, and following a thoracic stimulation (TTM and DLM pathway) in flies with constitutively and ubiquitously reduced insulin signaling. (**A**)Reduced IIS (da-GAL4/UAS-InR^dn^) prevented age-associated decline in the transmission through the DLM pathway (age x genotype interaction between the control genotypes and *da-GAL4/UAS-InR^dn^* is significant, P value < 0.0001; n = 6–16). (**B**) RLs to thoracic stimulation in young (7 days) and old (45 days) flies did not differ among genotypes and were ~40% shorter than RLs following brain stimulations (n = 6–7). Representative traces are on the right; vertical lines indicate the point to which response latency was measured. Both panels: error bars denote SEM.
(TIF)

S2 Fig Localization of shaking-B within the giant fiber system (GFS) (A), and anti-ChAT and anti-Dα7 Western blots (B). (**A**) *Left*: Merged confocal stacks of 5 days old Giant fibers (GF) injected with Rhodamine-dextran (magenta) and neurobiotin (white). Neurobiotin dye coupled to the peripheral synapsing interneurons (PSI) and TTM motoneurons. *Right*: 3D-reconstructed GFs (magenta) from left panel merged with 3D-reconstructed anti-SHAK-B staining (green) localizing to the GFs. Genotype: *A307-GAL4/+*. Scale bar: 8 μm. (**B**)Western blots of the CNS preparations from young (5–7 days) and old (45–50 days) flies probed with anti-ChAT antibody (n = 3), (*left*), anti-Dα7 antibody (n = 3–4) (*right*). Both panels: age x genotype interaction is not significant. Error bars denote SEM.
(TIF)

S3 Fig DLM response latencies and frequencies following an adult nervous system-specific IIS reduction. (**A**)Nervous-system-specific IIS down-regulation (RU+) prevented age-associated loss of transmission in the DLM branch (n = 7–10). (**B**)*Top-left*: Mean DLM response frequencies in middle aged flies during 5 sec following an air-puff stimulus. *Top-right*: Distribution of response frequencies from the left panel. RU(+) flies (red) showed higher response frequencies compared to RU(-) flies (blue) (RU (-): 15.8 Hz; RU(+): 27.3 Hz; n = 4). Each dot represents the average frequency of all responses between seconds 0 and 1, 1 and 2, etc. Bottom: Representative DLM responses. Both panels: error bars denote SEM.
(TIF)

S4 Fig Response latencies following a giant-fiber-specific IIS suppression, or *SHAK-B(N+16) *overexpression. (**A**)Giant fiber system-specific overexpression of *InR^dn^* abolished the age-related RL decline in the DLM branch of the Giant Fiber circuit (age x genotype interaction: P = 0.0001; n = 6–9 per genotype/age). (**B**)*split-GAL2* drives *InR^dn^* expression only in the Giant Fiber interneurons (interaction P value = 0.0463; n = 5–9 per genotype/age). (**C**)*Top*: Giant Fiber System-specific *SHAK-B(N+16)* over-expression prevented age-associated functional decline in the DLM part of the circuit in young (5–7 days) and old (45 days) flies (interaction P value = 0.015; n = 6–15 per genotype/age). *Bottom*: Representative DLM traces from 45 days old control *(left*) and *SHAK-B*-overexpressing (*right*) animals. Red arrows indicate RL periods. All panels: error bars denote SEM.
(TIF)

S5 Fig Giant fiber diameter measurements, *SHAK-B(N+16)* mRNA levels in old flies, and proteasomal activity in young and old flies. **(A) **Giant Fiber diameter measured in the first thoracic ganglion of old (~50 days) flies. *Left*: The GFs were injected with Rhodamine (red); yellow bars mark the positions where diameter measurements were taken. *Right*: Histogram of diameter measurements (n = 18–20). (**B**)Relative *SHAK-B(n+16)* mRNA levels in 45 days old flies with (*left*) ubiquitously and systemically lowered IIS (+control genotype), and (*right*) insulin signaling reduced only in the Giant Fiber System (+control genotype) (n = 3 per genotype). (**C**)Proteasomal activity in the heads of young (7 days) and old (40 days) flies. Age-associated reduction in chymotrypsin-like peptidase activity of the proteasome in fly heads (measured using the fluorogenic peptide substrate LLVY-AMC) was not attenuated in flies expressing a dominant-negative form of insulin receptor in the adult nervous system (*GS ELAV-GAL4/UAS*-*InR^dn^*, RU+) (n = 5–6).
(TIF)

S6 Fig Quantification of relevant total and cell surface protein levels in RPE1 cells. (**A**)Timelines of IIS manipulations. (**B **and **C**)Quantification from high-throughput microscopy images of the total levels of Cx43 in RPE1 cells grown in reduced IIS medium (‘Standard’) or insulin-free defined medium (‘Defined’) (Data shown as means +/- SEM from three independent experiments (over 12,000 Cx43 punctae per condition); *n*.*s*., not significant; ***P<0.001; one-way ANOVA and Dunnett’s test versus ‘-insulin’, or ‘0 insulin’, as appropriate. (**D**)Quantification from high-throughput microscopy images of cell surface levels of integrin α3 (‘ITGA3), Glut1 or Cx43 in RPE1 cells grown in reduced IIS medium (Data are normalized to respective levels in elevated IIS cells and shown as means +/- SEM from three independent experiments (over 12,000 Cx43 punctae per condition); *n*.*s*., not significant; ***P<0.001; one-way ANOVA and Dunnett’s test versus elevated IIS). (**E** and **F**)Quantification from flow cytometry acquisitions of the total Cx43 levels (E) or transferrin uptake (F) in RPE1 cells grown in reduced or elevated IIS conditions, and treated with NH_4_Cl and/or insulin, as indicated. (Data are shown as means +/- SEM from three independent experiments (over 10,000 cells per condition and per experiment); *n*.*s*., not significant; ***P<0.001; one-way ANOVA and Dunnett’s test.
(TIF)

S7 Fig DLM response latencies in flies with Rab4 or Rab11 levels manipulated in the GFS, shaking-B immunofluorescence area in young flies with reduced Rab4 or Rab11 levels, and the effect of Rab11 reduction in flies with attenuated IIS. (**A**)DLM response latencies from young (y, days 5–7) and old (o, days 45–50) flies, same as in Fig 4C (n = 4–8). (**B**)SHAK-B immunofluorescence area in the bilateral tracts of young (7 days old) flies (n = 8–10). (**C**)Response latency measured in ~45 day old flies. Rab11 is indispensable for the effect of reduced signaling on the conduction through the DLM branch of the GF circuit (n = 6–13). All panels: error bars denote SEM.
(TIF)

S1 Table Response latency data for all genotypes, RU conditions and pharmacological treatments. The *n* is denoted in figure legends.
(DOCX)

S1 Data Individual data points for all experiments. (XLSX)

## Abbreviations

BafA: bafilomycin A
CA: constitutively active
Cx43: connexin 43
DLM: dorsal longitudinal flight muscle
DLMn: dorsal longitudinal flight muscle motor neuron
DN: dominant-negative
GF: giant fiber
GFS: giant fiber system
GJ: gap junction
GLUT1: glucose transporter 1
IGF: insulin-like growth factor
IGF-1R: insulin-like growth factor-1 receptor
IIS: insulin/insulin-like growth factor signaling
IR: insulin receptor
ITGA3: integrin α3
LAMP1: lysosomal-associated membrane protein 1
nAChRs: nicotinic acetylcholine receptors
PKC: protein kinase C
PSI: peripherally synapsing interneurons
RPE1: retinal pigment epithelial
SHAK-B: shaking-B protein
TTM: tergotrochanteral muscle
TTMn: tergotrochanteral muscle motor neuron
WT: wild-type
