# Supplementary figures and images for: Reduced insulin signaling maintains electrical transmission in a neural circuit in aging flies
Source: PLoS Biol. 2017 Sep 13;15(9):e2001655. doi: 10.1371/journal.pbio.2001655 (PMC5597081; doi:10.1371/journal.pbio.2001655)

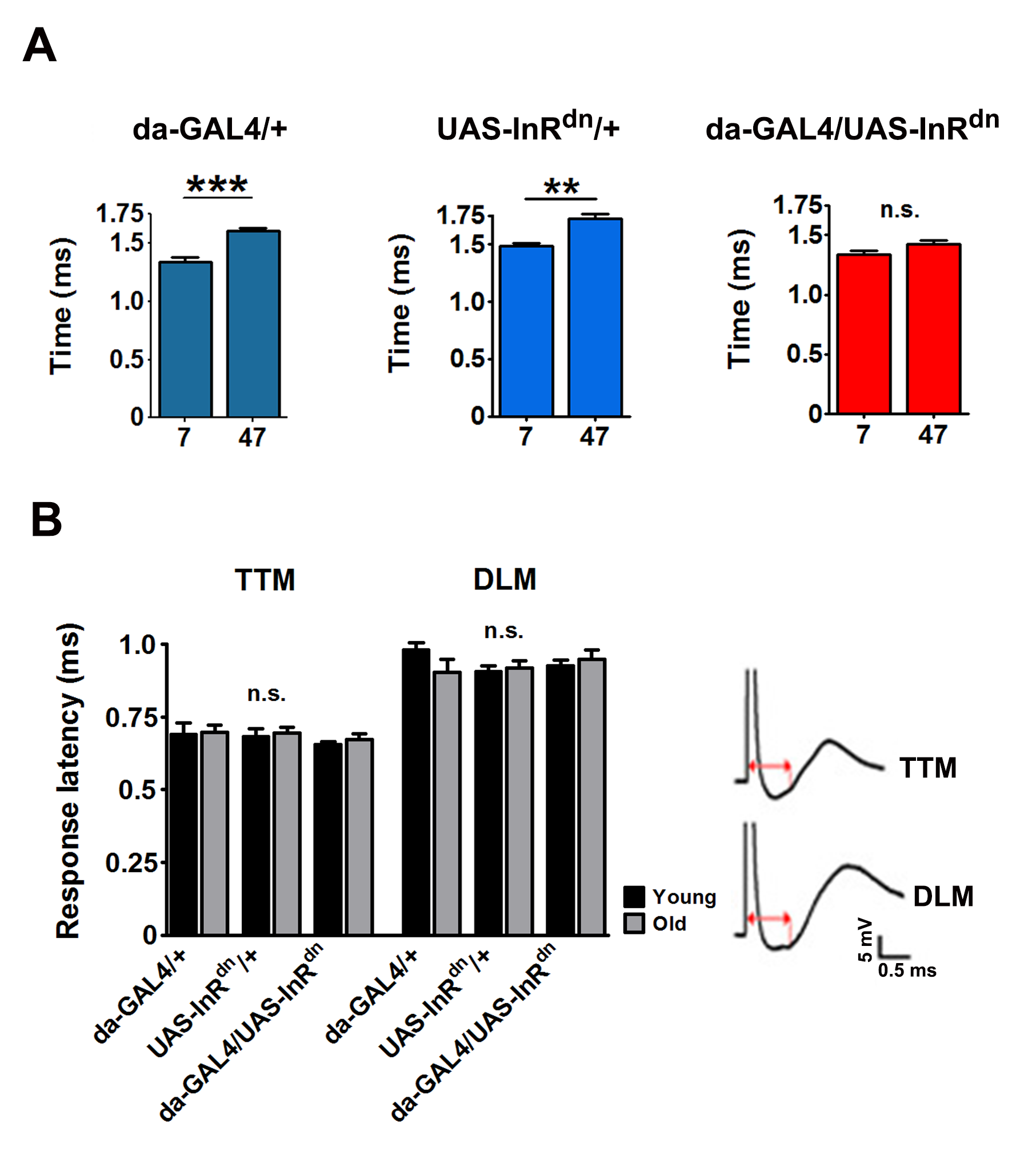

Supplement: S1 Fig — (A)Reduced IIS (da-GAL4/UAS-InRdn) prevented age-associated decline in the transmission through the DLM pathway (age x genotype interaction between the control genotypes and da-GAL4/UAS-InRdn is significant, P value < 0.0001; n = 6–16). (B) RLs to thoracic stimulation in young (7 days) and old (45 days) flies did not differ among genotypes and were ~40% shorter than RLs following brain stimulations (n = 6–7). Representative traces are on the right; vertical lines indicate the point to which response latency was measured. Both panels: error bars denote SEM. (TIF) [file pbio.2001655.s001.tif]

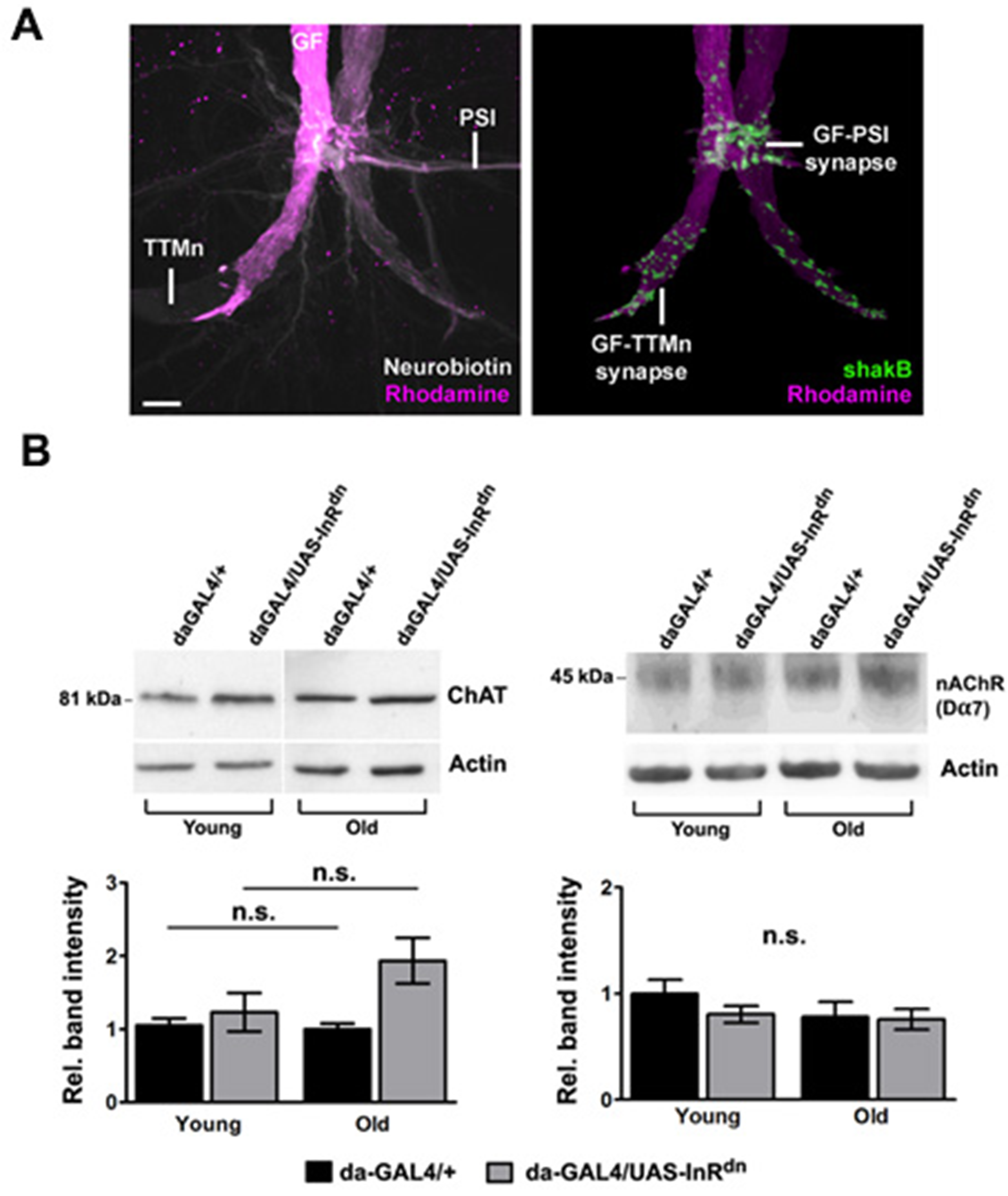

Supplement: S2 Fig — Localization of shaking-B within the giant fiber system (GFS) (A), and anti-ChAT and anti-Dα7 Western blots (B). (A) Left: Merged confocal stacks of 5 days old Giant fibers (GF) injected with Rhodamine-dextran (magenta) and neurobiotin (white). Neurobiotin dye coupled to the peripheral synapsing interneurons (PSI) and TTM motoneurons. Right: 3D-reconstructed GFs (magenta) from left panel merged with 3D-reconstructed anti-SHAK-B staining (green) localizing to the GFs. Genotype: A307-GAL4/+. Scale bar: 8 μm. (B)Western blots of the CNS preparations from young (5–7 days) and old (45–50 days) flies probed with anti-ChAT antibody (n = 3), (left), anti-Dα7 antibody (n = 3–4) (right). Both panels: age x genotype interaction is not significant. Error bars denote SEM. (TIF) [file pbio.2001655.s002.tif]

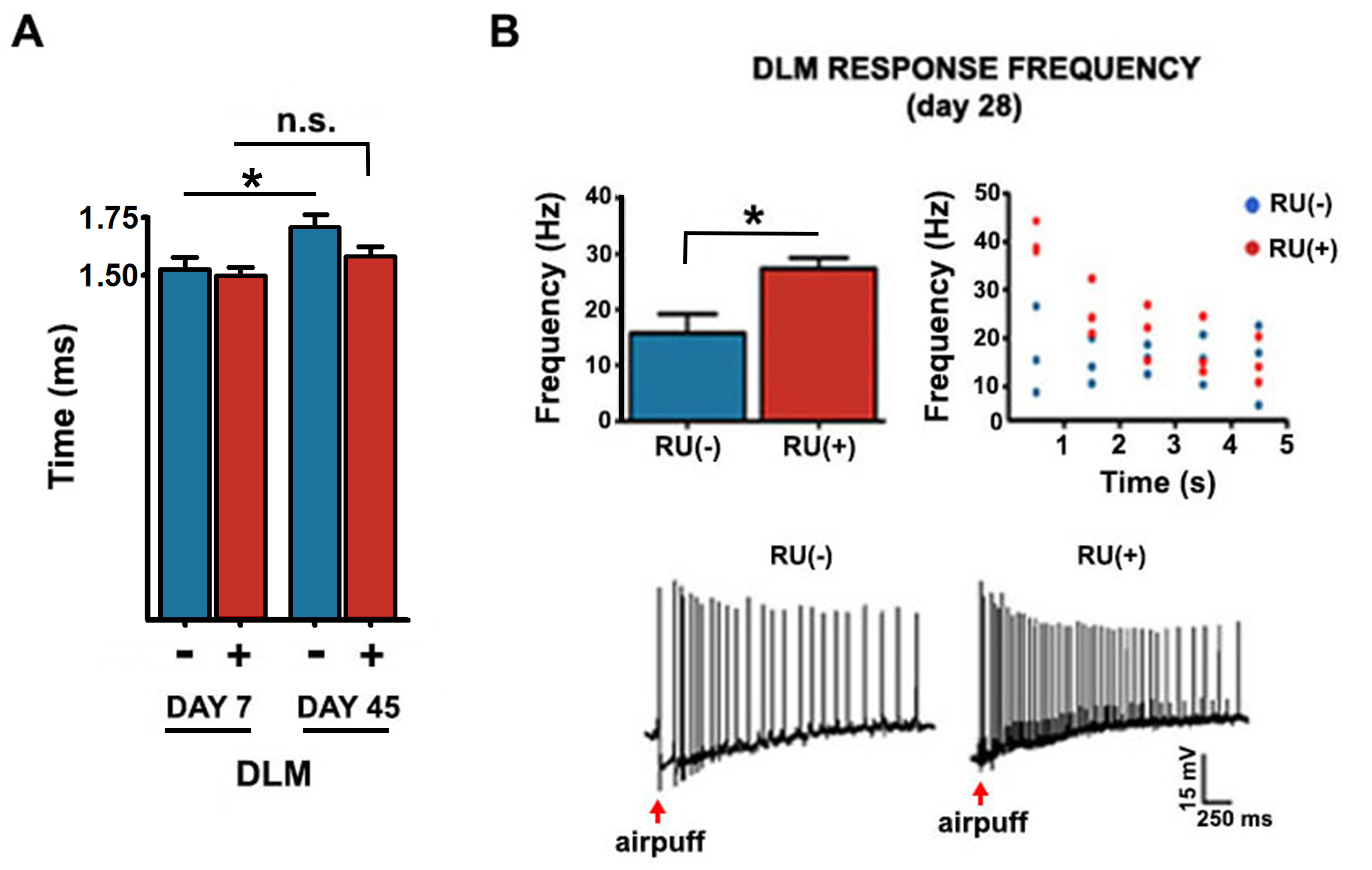

Supplement: S3 Fig — (A)Nervous-system-specific IIS down-regulation (RU+) prevented age-associated loss of transmission in the DLM branch (n = 7–10). (B)Top-left: Mean DLM response frequencies in middle aged flies during 5 sec following an air-puff stimulus. Top-right: Distribution of response frequencies from the left panel. RU(+) flies (red) showed higher response frequencies compared to RU(-) flies (blue) (RU (-): 15.8 Hz; RU(+): 27.3 Hz; n = 4). Each dot represents the average frequency of all responses between seconds 0 and 1, 1 and 2, etc. Bottom: Representative DLM responses. Both panels: error bars denote SEM. (TIF) [file pbio.2001655.s003.tif]

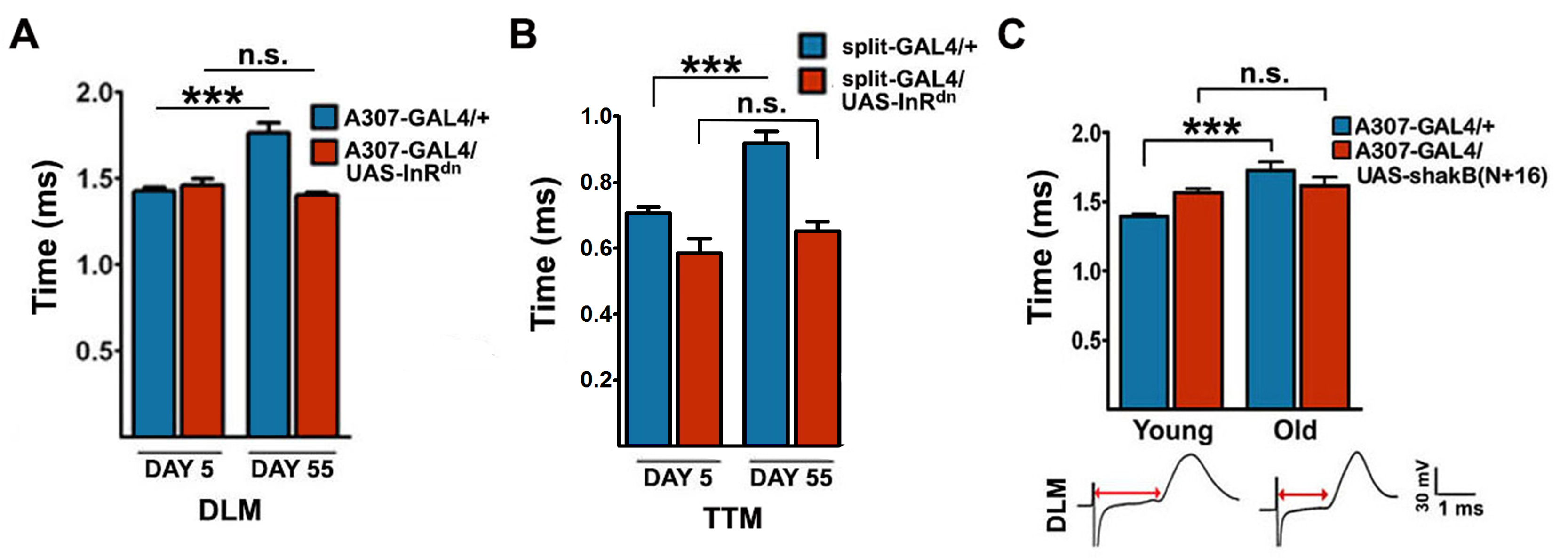

Supplement: S4 Fig — (A)Giant fiber system-specific overexpression of InRdn abolished the age-related RL decline in the DLM branch of the Giant Fiber circuit (age x genotype interaction: P = 0.0001; n = 6–9 per genotype/age). (B)split-GAL2 drives InRdn expression only in the Giant Fiber interneurons (interaction P value = 0.0463; n = 5–9 per genotype/age). (C)Top: Giant Fiber System-specific SHAK-B(N+16) over-expression prevented age-associated functional decline in the DLM part of the circuit in young (5–7 days) and old (45 days) flies (interaction P value = 0.015; n = 6–15 per genotype/age). Bottom: Representative DLM traces from 45 days old control (left) and SHAK-B-overexpressing (right) animals. Red arrows indicate RL periods. All panels: error bars denote SEM. (TIF) [file pbio.2001655.s004.tif]

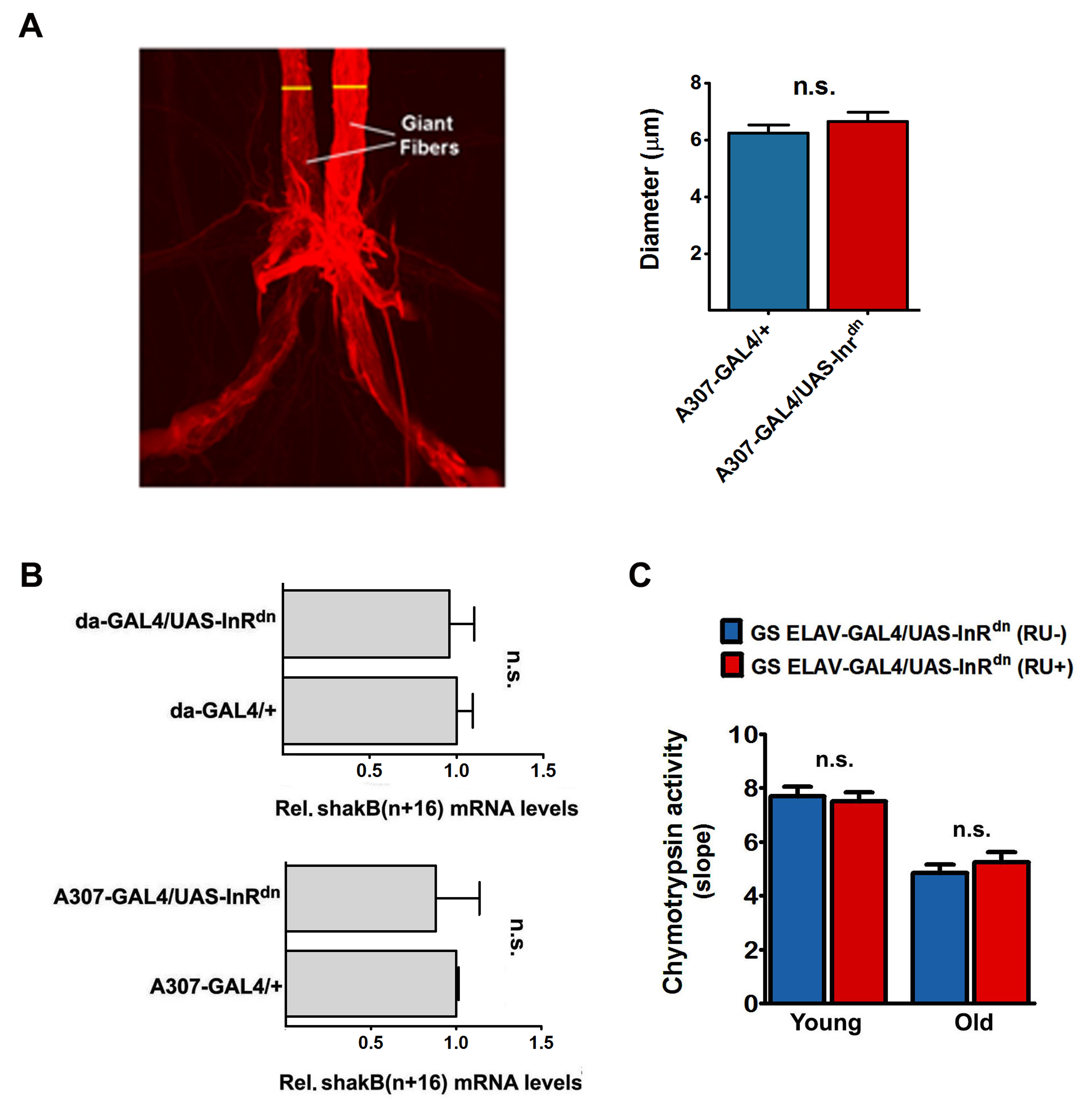

Supplement: S5 Fig — (A) Giant Fiber diameter measured in the first thoracic ganglion of old (~50 days) flies. Left: The GFs were injected with Rhodamine (red); yellow bars mark the positions where diameter measurements were taken. Right: Histogram of diameter measurements (n = 18–20). (B)Relative SHAK-B(n+16) mRNA levels in 45 days old flies with (left) ubiquitously and systemically lowered IIS (+control genotype), and (right) insulin signaling reduced only in the Giant Fiber System (+control genotype) (n = 3 per genotype). (C)Proteasomal activity in the heads of young (7 days) and old (40 days) flies. Age-associated reduction in chymotrypsin-like peptidase activity of the proteasome in fly heads (measured using the fluorogenic peptide substrate LLVY-AMC) was not attenuated in flies expressing a dominant-negative form of insulin receptor in the adult nervous system (GS ELAV-GAL4/UAS-InRdn, RU+) (n = 5–6). (TIF) [file pbio.2001655.s005.tif]

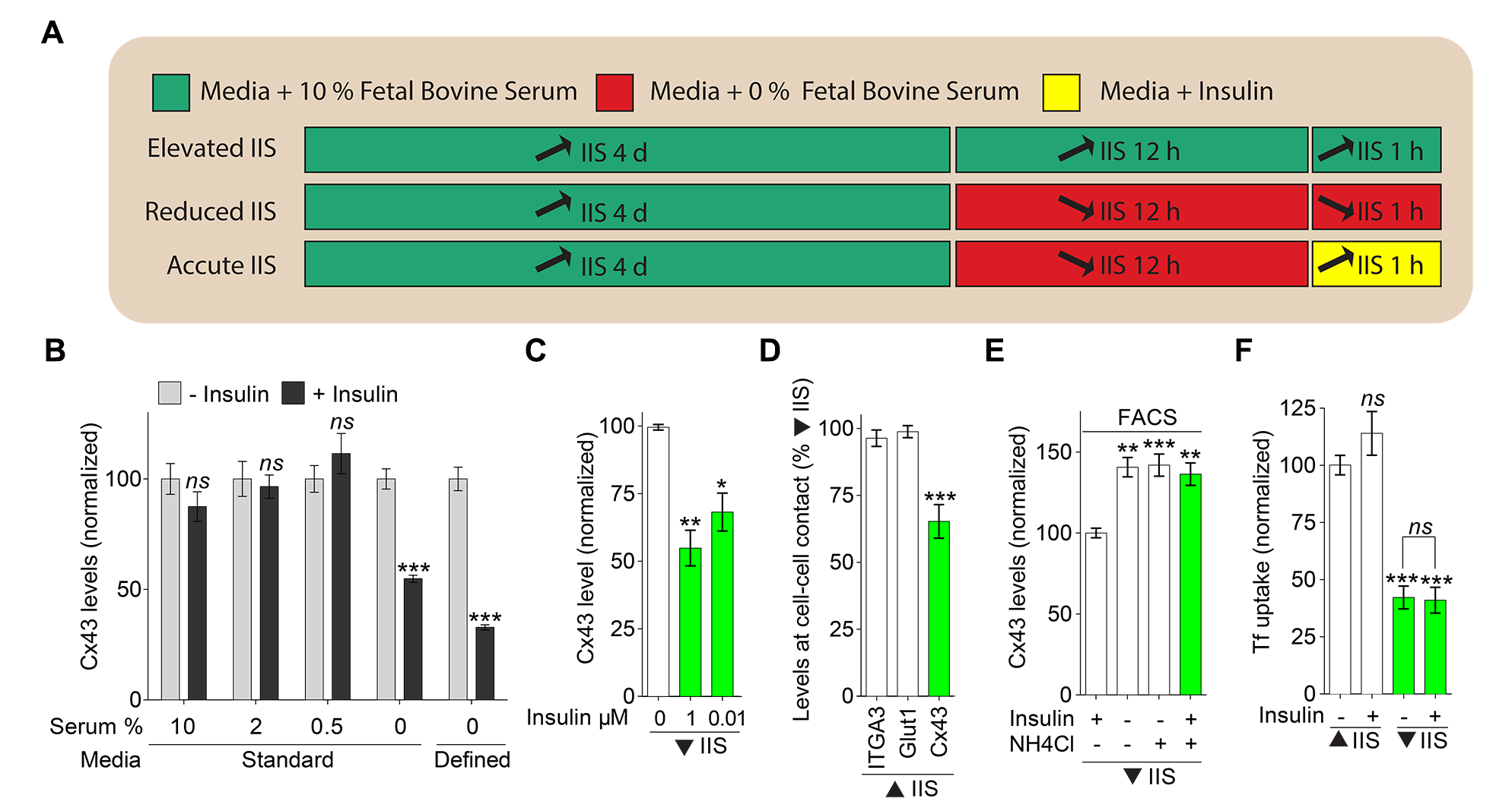

Supplement: S6 Fig — (A)Timelines of IIS manipulations. (B and C)Quantification from high-throughput microscopy images of the total levels of Cx43 in RPE1 cells grown in reduced IIS medium (‘Standard’) or insulin-free defined medium (‘Defined’) (Data shown as means +/- SEM from three independent experiments (over 12,000 Cx43 punctae per condition); n.s., not significant; ***P<0.001; one-way ANOVA and Dunnett’s test versus ‘-insulin’, or ‘0 insulin’, as appropriate. (D)Quantification from high-throughput microscopy images of cell surface levels of integrin α3 (‘ITGA3), Glut1 or Cx43 in RPE1 cells grown in reduced IIS medium (Data are normalized to respective levels in elevated IIS cells and shown as means +/- SEM from three independent experiments (over 12,000 Cx43 punctae per condition); n.s., not significant; ***P<0.001; one-way ANOVA and Dunnett’s test versus elevated IIS). (E and F)Quantification from flow cytometry acquisitions of the total Cx43 levels (E) or transferrin uptake (F) in RPE1 cells grown in reduced or elevated IIS conditions, and treated with NH4Cl and/or insulin, as indicated. (Data are shown as means +/- SEM from three independent experiments (over 10,000 cells per condition and per experiment); n.s., not significant; ***P<0.001; one-way ANOVA and Dunnett’s test. (TIF) [file pbio.2001655.s006.tif]

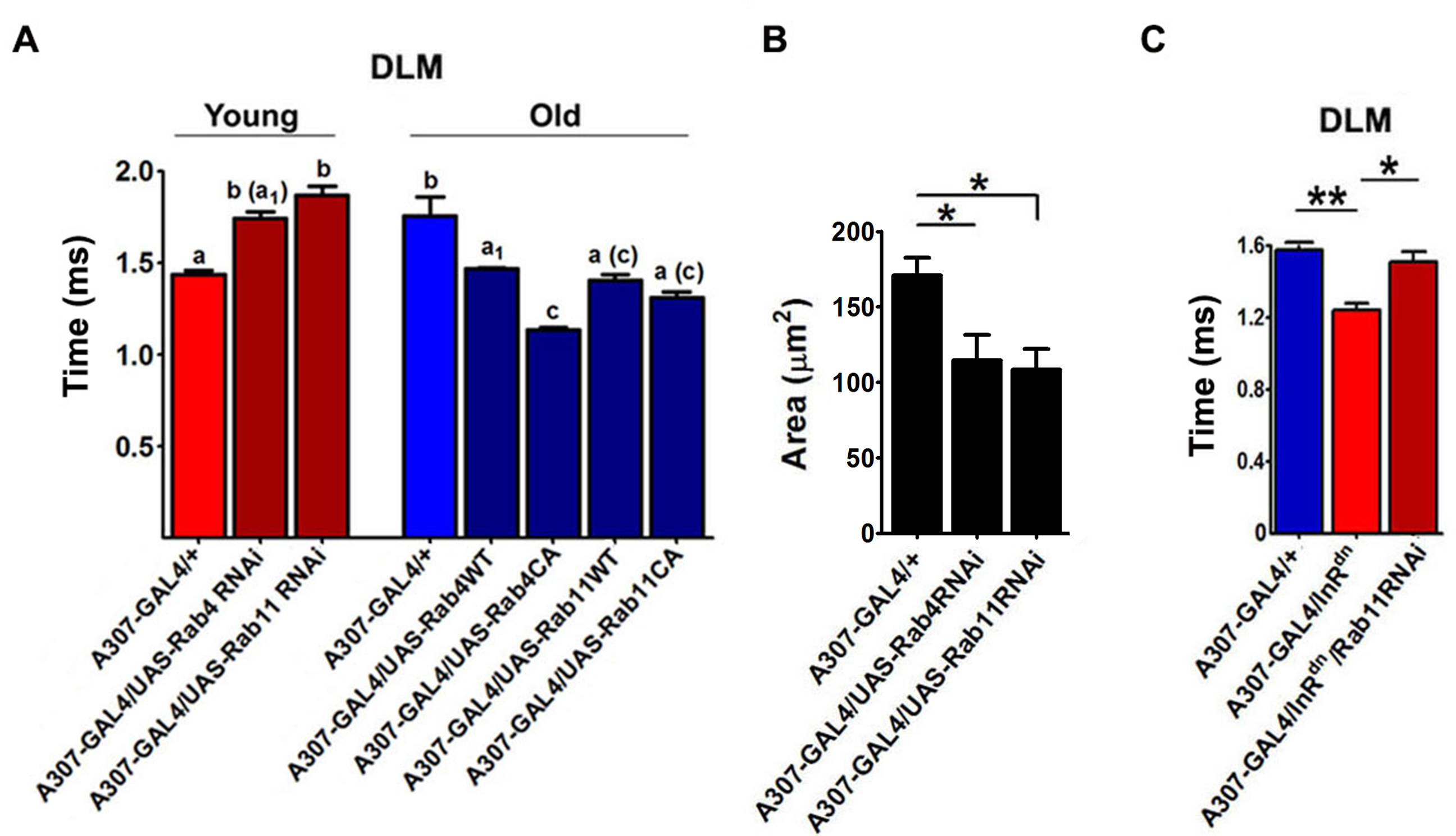

Supplement: S7 Fig — (A)DLM response latencies from young (y, days 5–7) and old (o, days 45–50) flies, same as in Fig 4C (n = 4–8). (B)SHAK-B immunofluorescence area in the bilateral tracts of young (7 days old) flies (n = 8–10). (C)Response latency measured in ~45 day old flies. Rab11 is indispensable for the effect of reduced signaling on the conduction through the DLM branch of the GF circuit (n = 6–13). All panels: error bars denote SEM. (TIF) [file pbio.2001655.s007.tif]
