# Supplementary material for: Reduced insulin signaling maintains electrical transmission in a neural circuit in aging flies
Source: PLoS Biol. 2017 Sep 13;15(9):e2001655. doi: 10.1371/journal.pbio.2001655 (PMC5597081; doi:10.1371/journal.pbio.2001655)
Supplement: S1 Table — The n is denoted in figure legends. (DOCX) [file pbio.2001655.s008.docx]

**Table S1. Response latency data for all genotypes, RU conditions and pharmacological treatments.** The *n* is denoted in figure legends.

| **FIG.** | **GENOTYPE** | **TTM RL (mean +/- SEM) in ms** | | | **DLM RL (mean +/- SEM) in ms** | | |
| --- | --- | --- | --- | --- | --- | --- | --- |
|  |  | **Young**  **(d 5-7)** | **Mid- agEd**  **(d25**  **27)** | **Old**  **(d45-50)** | **Young**  **(d 5-7)** | **MiD-aged**  **(d25-27)** | **Old**  **(d45-50)** |
| **1B** | ***WT (w^Dah^)*** | **0.926** **± 0.03** | **1.097 ± 0.03** | **1.216 ± 0.03** | **1.440 ± 0.04** | **1.593 ± 0.04** | **1.849 ± 0.03** |
| **1C, S1A** | ***da-GAL4/+*** | **0.937 ± 0.03** | **-** | **1.079 ± 0.04** | **1.335 ± 0.05** | - | **1.610 ± 0.02** |
|  | ***UAS-InR^dn^/+*** | **1.031 ± 0.02** | **-** | **1.154 ± 0.03** | **1.485 ± 0.02** | - | **1.722 ± 0.04** |
|  | ***daGAL4/UAS-InR^dn^*** | **0.907 ± 0.02** | **-** | **0.895 ± 0.03** | **1.340 ± 0.03** | - | **1.422 ± 0.04** |
| **S1B** | ***da-GAL4/+*** | **0.689 ± 0.04** | **-** | **0.692 ± 0.02** | **0.981 ± 0.04** | **-** | **0.904 ± 0.04** |
|  | ***UAS-InR^dn^/+*** | **0.683 ± 0.03** | **-** | **0.695 ± 0.02** | **0.905 ± 0.02** | **-** | **0.917 ± 0.03** |
|  | ***daGAL4/UAS-***  ***InR^dn^*** | **0.655 ± 0.01** | **-** | **0.673 ± 0.02** | **0.925 ± 0.02** | **-** | **0.949 ± 0.03** |
| **3A,**  **S3A** | ***GS ELAV***  ***GAL4/UAS***  ***InR^dn^ (-RU)*** | **1.027 ± 0.02** | **-** | **1.152 ± 0.03** | **1.525 ± 0.05** | **-** | **1.706 ± 0.05** |
|  | ***GS ELAV***  ***GAL4/UAS-InR^dn^ (+RU)*** | **1.076 ± 0.03** | **-** | **1.087 ± 0.02** | **1.495 ± 0.04** | **-** | **1.579 ± 0.04** |
| **3B** | ***GS ELAV***  ***GAL4/UAS-InR (-RU)*** | **0.933 ± 0.03** | **-** | **1.087 ± 0.07**  **(day 35)** | **-** | **-** | **-** |
|  | ***GS ELAV-GAL4/UAS-InR (+RU)*** | **0.987 ± 0.02** | **-** | **1.384 ± 0.06**  **(day 35)** | **-** | **-** | **-** |
| **4A, S4A** | ***A307-GAL4/+*** | **0.986 ± 0.05** | **-** | **1.180 ± 0.04**  **(day 55)** | **1.465 ± 0.04** | **-** | **1.768 ± 0.05 (day 55)** |
|  | ***A307-GAL4/ UAS-InR^dn^*** | **1.017 ± 0.03** | **-** | **0.928 ± 0.03**  **(day 5)** | **1.459 ± 0.04** | **-** | **1.389 ± 0.01**  **(day 55)** |
| **S4B** | ***split-GAL4/+*** | **0.706 ± 0.02** | **-** | **0.919 ± 0.04** | **-** | **-** | **-** |
|  | ***split-GAL4/ UAS-InR^dn^*** | **0.585 ± 0.04** | **-** | **0.652 ± 0.03** | **-** | **-** | **-** |
| **4D, S4C** | ***A307-GAL4/+*** | **0.967 ± 0.02** | **-** | **1.235 ± 0.09** | **1.395 ± 0.02** | **-** | **1.759 ± 0.10** |
|  | ***A307-GAL4/ UAS-SHAK-B(N+16)*** | **0.997 ± 0.03** | **-** | **0.977 ± 0.06** | **1.566 ± 0.03** | **-** | **1.614 ± 0.06** |
| **6C, S7A** | ***A307-GAL4/+*** | **0.969 ± 0.01** | **-** | **1.235 ± 0.10** | **1.438 ± 0.02** | **-** | **1.759 ± 0.10** |
|  | ***A307-GAL4/ UAS-Rab4RNAi*** | **1.177 ± 0.03** | **-** | **-** | **1.743 ± 0.04** | **-** | **-** |
|  | ***A307-GAL4/ UAS-Rab11RNAi*** | **1.672 ± 0.04** | **-** | **-** | **1.871 ± 0.05** | **-** | **-** |
|  | ***A307-GAL4/ UAS-Rab4WT*** | **-** | **-** | **1.005 ± 0.02** | **-** | **-** | **1.468 ± 0.01** |
|  | ***A307-GAL4/ UAS-Rab4CA*** | **-** | **-** | **0.792 ± 0.01** | - | **-** | **1.137 ± 0.04** |
|  | ***A307-GAL4/ UAS-Rab11WT*** | **-** | **-** | **0.831 ± 0.02** | - | **-** | **1.407 ± 0.03** |
|  | ***A307-GAL4/ UAS-Rab11CA*** | **-** | **-** | **0.899 ± 0.03** | **-** | **-** | **1.312 ± 0.03** |
| **6E, S7C** | ***A307-GAL4/+*** | **-** | **-** | **1.095 ± 0.04** | **-** | **-** | **1.577 ± 0.07** |
|  | ***A307-GAL4/UAS-InR^dn^*** | **-** | **-** | **0.801 ± 0.08** | **-** | **-** | **1.240 ± 0.04** |
|  | ***A307-GAL4/ UAS-InR^dn^/ Rab11-RNAi*** | **-** | **-** | **1.110 ± 0.06** | **-** | **-** | **1.511 ± 0.06** |
